# Nonmimetic Gels Direct Novel Crystallization Behavior of Lenalidomide

**DOI:** 10.1021/acs.cgd.5c01083

**Published:** 2025-09-25

**Authors:** Martin A. Screen, Juan A. Aguilar, Toby J. Blundell, James F. McCabe, Sean Askin, Clare S. Mahon, Mark R. Wilson, Jonathan W. Steed

**Affiliations:** 1 Department of Chemistry, 3057 Durham University, South Road, Durham DH1 3LE, United Kingdom; 2 Early Pharmaceutical Development & Manufacture, Pharmaceutical Sciences, R&D, AstraZeneca, Macclesfield SK10 2NA, United Kingdom; 3 Advanced Drug Delivery, Pharmaceutical Sciences, R&D, AstraZeneca, Cambridge CB2 0AA, United Kingdom

## Abstract

Crystallization within supramolecular gels can yield distinct solid-state outcomes compared with conventional solution-phase methods, including the formation of novel crystal forms or selectively crystallizing one crystal form from a concomitant mixture. In several cases, tailoring the molecular structure of a gelator to mimic a pharmaceutical substrate has facilitated crystallization control, where nonmimetic gelators had no influence on the crystallization outcome compared to the solution phase. In this study, we investigate the crystallization behavior of lenalidomide within both mimetic and nonmimetic gels. Crystallization in a cyclopentanone gel using a nonmimetic gelator led to the discovery of a novel cyclopentanone hemisolvate, inaccessible via solution-phase crystallization. Additionally, an ethanol gel of the same gelator promoted selective crystallization of metastable Form 4 in ethanol, in contrast to the thermodynamically favored Form 1 obtained from solution. Gel-phase crystallization using a drug-mimetic gelator produced no deviation from the solution-phase polymorphic outcomes, in contrast to previously reported examples. Solution-state NMR studies showed no evidence of strong interactions between lenalidomide and either gelator, suggesting that the spatial arrangement of the nonmimetic gel fibers and/or possible confinement effects, rather than solution association, plays a critical role in directing crystallization behavior.

## Graphical abstract

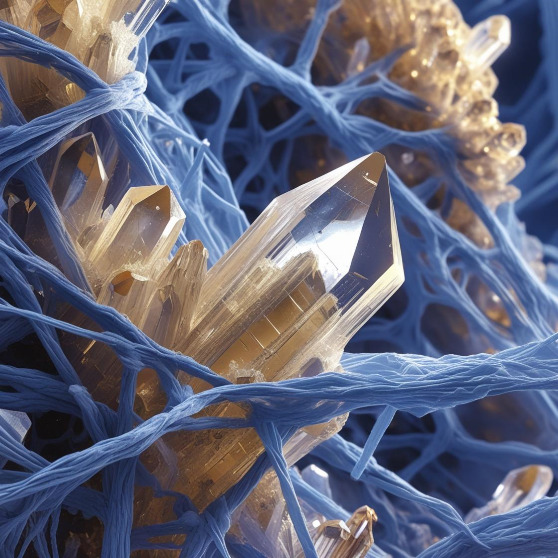

## Introduction

Understanding and controlling the solid-state characteristics of a drug substance are critical to ensure that drug products exhibit adequate bioavailability and maintain both physical and chemical stability. Industrial solid-form screening reveals that more than half of active pharmaceutical ingredients (APIs) are polymorphic,^1^ meaning that they can adopt multiple crystalline forms due to variations in intermolecular arrangements. Polymorphism presents both potential benefits and complications to the pharmaceutical industry: while it enables the selection of solid forms with desirable properties, such as enhanced bioavailability, it also poses a risk of unwanted phase transformations that can compromise the drug’s performance.^2,3^ The physicochemical properties of different solid forms—ranging from anhydrous polymorphs and solvates to salts and amorphous phases—can vary significantly, particularly in their solubility and physical stability.^4^ Control of particle size and crystal morphology is also crucial in addition to solid form selection, as these factors significantly influence drug dissolution, manufacturability, and overall performance.^5^ As such, a comprehensive exploration of API crystallization is essential for achieving a consistent and reliable pharmaceutical performance in new drug candidates. While conventional solution-phase crystallization remains the predominant approach used in industry, crystallization in gels has also proven effective in directing and controlling drug polymorphic outcomes.^6,7^ Gel-phase crystallization approaches can produce different polymorphic or morphology outcomes to solution-phase methods in the same solvents because the gel network inhibits convection and sedimentation, allowing the diffusion-limited growth of the crystallization substrate.^6,8,9^ The gel fibers themselves may also act as an active surface for heterogeneous secondary nucleation, where the underlying periodicity of the gel fibers arising from aggregation may directly influence the growth of the crystal.^10^ This has allowed the discovery of new solid forms and unique crystal morphologies,^7,10,11^ as well as preventing concomitant crystallization and enabling the selective growth of desired polymorphs.^9,12–16^

Supramolecular gels are comprised of low-molecular-weight gelators (LMWGs) that form gel fibers via self-assembly.^17–20^ Bis­(urea) compounds are some of the most explored LMWGs since they are cheap, easy to prepare, and can gel a wide range of solvents, providing a greater variety of gel phases in which drug crystallization can be screened in comparison to conventional polymeric hydrogels.^7,21,22^ LMWGs such as these form physical gels reversibly via noncovalent interactions such as hydrogen-bonding, π-stacking, van der Waals forces, charge transfer interactions, electrostatic interactions, and metal coordination, meaning that many supramolecular gels are stimuli responsive and can be redissolved in situ by addition of anions, change in pH, sonication, or exposure to light to enable simple isolation of the crystallized substrate.^23–29^ Bis­(urea) gelation in particular is driven by molecular recognition of the self-complementary urea moiety, where the ability to simultaneously donate hydrogen-bonds through two NH protons and accept hydrogen-bonds through lone pairs on the carbonyl oxygen allows the formation of bifurcated intermolecular hydrogen-bonds between urea groups, known as a urea α-tape motif.^30^ The structure of LMWGs can also be designed to mimic the crystallization substrate by including similar moieties in the gelator end-group functionalities: there are numerous examples of drug-mimetic bis­(urea) gelators capable of producing unique crystallization outcomes that could not be obtained from the equivalent solution phase nor from gel-phase crystallization where the gelator was nonmimetic. We have previously shown that the metastable red form of ROY can be crystallized within gels of a drug-mimetic bis­(urea) compound whereas only the thermodynamic stable yellow form could be obtained from solution or using nonmimetic gelators and proposed that the red form may have been templated by conformational matching with the gelator in the fibers.^8^ We also found that the concomitant crystallization of thalidomide Forms α and β and of barbital Forms I, III, and V observed by crystallization in either the solution-phase or within gels of nonmimetic gelators could only be prevented by crystallizing within gels of drug-mimetic bis­(urea) gelators, indicating that the drug-mimetic structure was responsible for the crystallization control rather than the viscous gel media itself.^9^ Mimetic gels have also revealed a new dimethylacetamide solvate of cisplatin and given rise to a new crystal habit of the known dimethylformamide solvate by crystallization in bis­(urea) gelators, where the only crystals suitable for analysis by single-crystal X-ray diffraction (SC-XRD) were obtained using the drug-mimetic gelator.^10^ Similarly, the crystal habit of metronidazole crystals can be controlled within gels of a drug-mimetic gelator, yet not in those of a nonmimetic gelator.^11^ The thermodynamic polymorph of flufenamic acid can be obtained by crystallization within gels of a pH-responsive drug-mimetic bis­(amide) gelator, whereas only the metastable form can be obtained from solution-phase crystallization.^12^ Finally, the highest energy Form 2 of mexiletine hydrochloride, usually only stable at high temperature, can be crystallized within gels of a drug-mimetic bis­(urea) gelator and the metastable Form 3 can be produced selectively in gels over the concomitant mixture with Form 1.^13^

Lenalidomide (LDM) is an immunomodulatory drug used for the treatment of multiple myeloma and pulmonary fibrosis, similar in structure to thalidomide. Celgene originally reported eight polymorphic and solvate forms termed Forms A–H, including the commercially available hemihydrate, Form B, marketed under the trade name Revlimid.^31^ In 2017, Chennuru et al. reported the single-crystal structures of seven polymorphs, hydrates, and solvates termed Forms 1–7 of which Forms 1, 2, and 7 are the same as Celgene Forms A, B, and E (Table 1).^31,32^ In addition, further three forms termed α, β, and DH have been reported, although not structurally characterized, and it is not clear whether these represent genuinely new materials.^33^ There are also reports of cocrystals with urea, 3,5-dihydroxybenzoic acid, acesulfamate, nicotinamide, and melamine.^34–38^ Despite the rich solid-form landscape already revealed through several extensive solid-form screening studies, it is possible that new crystalline forms of LDM may be accessible from gel-phase crystallization or that hard-to-access polymorphs such as the metastable Form 4, which can be accessed only by dehydrating one of the LDM hydrate forms, could be crystallized directly within gel media. In this work, we present the gel-phase crystallization of LDM within gels of both a drug-mimetic gelator and a nonmimetic gelator to study the importance of structural resemblance between drug and gelator in determining the crystallization outcome.

**Table 1:** Nomenclature and Details of LDM Crystal Forms with Structures in the CSD^a^

**LDM crystal form**	**details**
Form 1, Form A	anhydrous, thermodynamic
Form 2, Form B	hemihydrate
Form 3	DMF solvate
Form 4	anhydrous, metastable
Form 5	DMSO solvate
Form 6, Form C	acetone solvate
Form 7, Form E	dihydrate

a Forms 1–7 are designated by Chennuru et al.,^32^ whereas the original patents used lettering A–H to distinguish between forms.^31^

## Experimental Section

### Materials and General Methods

Lenalidomide (LDM, racemic form), l-alanine methyl ester hydrochloride, triethylamine, 1,6-diisocyanatohexane, D,L-aminoglutethimide, and 4,4′-methylenebis­(2,6-diethylphenyl isocyanate) were purchased from Merck. All other chemicals and solvents were available from commercial sources and used without further purification. FTIR spectra were recorded between 4000 and 550 cm^–1^ by using a PerkinElmer 100 FT-IR spectrometer with a μATR attachment. Powder X-ray diffraction (XRPD) patterns were collected at room temperature using a Bruker AXS D8 Advance GX003410 diffractometer with a Lynxeye Soller PSD detector, using Cu Kα radiation at a wavelength of 1.5406 Å and collecting from 2° ≤ 2θ ≤ 40°. Nuclear magnetic resonance (NMR) spectra were recorded on a Bruker Neo-400 spectrometer with operating frequencies of 400.20 MHz for ^1^H and 100.63 MHz for ^13^C unless otherwise specified. Mass spectrometry was performed using a Waters Acquity SQD machine running in positive electron spray (ES) mode. Elemental analysis was performed using an Exeter Analytical Inc. CE-400 elemental analyzer.

### Gelator Syntheses

Gelators were prepared using the previously published procedures for nonmimetic gelator G1 ^39^ and imide-mimetic gelator G2.^9^ Full procedures and characterization are provided in the Supporting Information.

### Gel Screening

The gelation behavior of compounds G1 and G2 was analyzed in a range of solvents suitable for crystallizing LDM. Gelator solids and solvents were added to vials and gently heated to dissolve them at an initial concentration of 1% w/v. Gel formation was typically observed several minutes after cooling to room temperature and confirmed in the first instance by a qualitative vial inversion test. Some samples formed only weak gels or viscous liquids on cooling, which failed the inversion test but produced gels after heating again to redissolve followed by 30 s of sonication. Samples that dissolved but precipitated upon cooling were repeated at 0.5% w/v. Insoluble samples at 1% w/v were not studied further. Samples that formed gels were repeated at progressively lower concentrations in 0.1% w/v increments until only a partial gel formed, characterized either by the gel being too weak to pass the inversion test or by the presence of some ungelled solvent remaining, to determine the critical gelation concentration (CGC). Samples increasing in concentration by the same 0.1% (w/v) increments were also produced to determine the maximum gelation concentration (MGC).

### Crystallization Studies of LDM

The solubility of LDM in a range of 24 solvents varying in polarity and boiling point was assessed at room temperature using a gravimetric method. An excess of LDM powder was stirred as a slurry in each solvent for 24 h using an Expondo roller mixer at 100 rpm, before filtering and transferring an accurately measured volume of supernatant (1 mL) to a preweighed vial and leaving to evaporate in an oven at 200 °C overnight before weighing again to determine the dissolved concentration of LDM. Solubility data are listed in Supporting Information Table S1. The crystallization of LDM was then studied in a range of ten solvents in which LDM has a solubility of at least 1% w/v and which can be gelled by gelator G1 and/or G2, by passive cooling of hot solutions prepared at 1.2 times the measured solubility of LDM at room temperature. Polymorphic outcome and phase purity were assessed by XRPD and SC-XRD, and crystal growth time and morphology were assessed by optical microscopy.

### Gel-Phase Crystallization Studies of LDM

Gel-phase recrystallization experiments were conducted by adding LDM and gelator powders to vials followed by dilution with solvents such that the resulting concentration of LDM was 1.2 times the measured solubility of LDM at room temperature and the resulting concentration of gelator was at either the CGC or MGC for each solvent. Control crystallization experiments of LDM in the absence of a gelator were set up at the same time. The samples were left undisturbed for 2 weeks prior to XRPD and SC-XRD analysis or until crystals were visible. XRPD slides were prepared by transferring samples of the gel-crystal mixtures by using a spatula and leaving them to dry by evaporation prior to analysis. Where possible, the polymorphic outcome was confirmed with unit cell determination by single-crystal X-ray diffraction. Gel-phase crystallization experiments that differed in outcome from the control experiment were repeated in triplicate. Crystallization experiments within blended gels were also conducted using the same method but consisting of varying molar ratios of both G1 and G2 (molar ratios of 2:8, 4:6, 6:4, and 8:2) in both dioxane and cyclopentanone.

### Rheometry

Cyclopentanone gels of G1 and G2, at both CGC and MGC, and both with and without LDM crystals, were analyzed by oscillatory rheometry. Rheological experiments were performed using an advanced rheometer AR 2000 from TA Instruments equipped with a chiller and using stainless steel 20 mm parallel plate geometry. Samples of the gels were transferred on to the center of the rheometer plate using a spatula. The oscillatory stress–sweep measurements were performed in a range of 0.1–100 Pa at a constant frequency of 1 Hz. Frequency sweep measurements were performed in a range of 1–10 rad/s at a constant oscillatory stress of 0.5 Pa, within the linear viscoelastic region of the gels analyzed.

### Scanning Electron Microscopy (SEM)

SEM samples of G1 and G2 cyclopentanone xerogels, both with and without LDM crystals, were prepared by adding solid powders to polycarbonate wafers and coating them with 5 nm of platinum using a Cressington 328 Ultra High-Resolution EM Coating System. The images were obtained using a Carl Zeiss Sigma 300 VP FEG SEM microscope operated at 5 kV using an in-lens detector.

### Single-Crystal X-ray Diffraction (SCXRD)

Single-crystal X-ray diffraction data for LDM Form 8 and G1 Form B were collected at 120 K using Mo Kα radiation at a wavelength of 0.71073 Å on a Bruker D8 Venture 3-circle diffractometer. The structures were solved by direct methods and refined by full-matrix least-squares on *F* ^2^ for all data using Olex2 ^40^ and SHELXTL.^41^ All nonhydrogen atoms were refined in anisotropic approximation, while hydrogen atoms were placed in the calculated positions and refined in riding mode unless otherwise specified. Crystal data for LDM Form 8: C_15.5_H_17_N_3_O_3.5_, *M*_r_ = 301.32, space group *P* 1̅1̅ , *a* = 11.1851(7) Å, *b* = 12.1666(8) Å, *c* = 12.6313(8) Å, α = 62.187(2)°, β = 78.555(2)°, γ = 66.633(2)°, *V* = 1395.46(16) Å^3^, *R*_1_ (*I* > 2σ­(*I*)) = 0.0574, *wR*_2_ (all data) = 0.1243. Full crystallographic data, parameters of refinement, and hydrogen-bonding distances and angles are listed in Supporting Information Tables S2 and S3. The structure was deposited in the CCDC under deposition number 2453375. Crystal data for G1 Form B: C_16_H_30_N_4_O_6_, *M*_r_ = 374.44, space group *P*1, *a* = 4.6439(4) Å, *b* = 6.0527(5) Å, *c* = 17.9322(15) Å, α = 95.476(3)°, β = 94.920(3)°, γ = 108.854(3)°, *V* = 471.17(7) Å^3^, *R*_1_ (*I* > 2σ­(*I*)) = 0.0521, *wR*_2_ (all data) = 0.1231. Full crystallographic data, parameters of refinement, and the hydrogen-bonding distances and angles are listed in Supporting Information Tables S4 and S5. The structure was deposited in the CCDC under deposition number 2453376.

### NMR Titrations

^1^H NMR spectra were recorded on a Varian DD2–500 spectrometer with an operating frequency of 499.53 MHz. Gelator solutions at a concentration of 1.4 mg/mL (below CGC) in dioxane-*d*_8_ were analyzed at 90 °C before sequentially adding aliquots of a lenalidomide solution in dioxane-*d*_8_ and reacquiring the spectra such that the concentration of lenalidomide doubled between consecutive ^1^H spectra. This was repeated until eight scans had been acquired for each gelator, ranging from a gelator:drug molar ratio of 1:0.04 up to 4.5:1, near the solubility limit of LDM in dioxane. Chemical shifts for lenalidomide and the gelators were monitored throughout the titration.

### Diffused Ordered NMR Spectroscopy (DOSY)

A 600 MHz Varian spectrometer equipped with an Agilent OneNMR Probe able to deliver a maximum pulsed field gradient of 62 G cm^–1^ was used to conduct diffusiometry studies (^1^H Diffusion-Ordered SpectroscopY or DOSY) using a convection-compensated pulse sequence based on a combination of a double stimulated echo and bipolar pulsed gradients. The pulse sequence, Dbppstee_cc, is part of the VNMJ 4.2 pulse sequence library.^42,43^ Twenty gradient amplitudes ranging from 1.95 to 29.25 G cm^–1^ spaced in equal steps of gradient squared were used. Thirty-two transients were collected. Thirty-two steady-state transients were used. The number of complex data points was 21,406, covering 6.3 kHz. The diffusion-encoding pulsed gradient duration (*d*) was 2.0 ms. The diffusion time was (*D*) 200 ms. The gradient stabilization delay was 2.0 ms. The repetition time was 6.4 s, of which 3.4 s comprised the acquisition time. The unbalancing factor was 0.15. The results were analyzed with VNMRJ 4.2 using monoexponential fittings. The effects of nonuniform field gradients were accounted for using methods developed by Morris et al.^44^

## Results and Discussion

### Gel Screening and Characterization

Two bis­(urea) gelator compounds, **G1** and **G2** (Scheme 1), were chosen for gel-phase crystallization studies of lenalidomide (LDM). **G1** is a representative nonmimetic gelator bearing no structural resemblance to LDM, whereas **G2** is an imide-based drug-mimetic gelator shown previously to control the concomitant crystallization of thalidomide, which is closely related to LDM.^9,39^ Comparing the crystallization behavior of LDM within gels of these two compounds provides an opportunity to understand how important drug–gelator structural similarity is for controlling drug crystallization in the gel phase. **G1** and **G2** were synthesized via the previously reported methodologies. The observed gelation behavior of **G1** and **G2** differs somewhat from our previously reported results, particularly for **G2,** which appears to gel fewer solvents than originally reported. This may be caused by trace impurities from synthesis, differences in moisture content, or because the reagents were obtained from different suppliers. Table 2 shows the gelation results along with critical gelation concentrations (CGCs) and maximum gelation concentrations (MGCs) for **G1** and **G2** over a range of organic solvents. **G1** is capable of gelling eight solvents with CGCs between 0.5 and 1% w/v, while **G2** is capable of gelling six solvents with CGCs between 0.1 and 2% w/v. Cyclopentanone, nitrobenzene, and 1,4-dioxane can be gelled by both compounds at similar gelator concentrations, allowing for a direct comparison of the gel-phase recrystallization of LDM between three gel-phase systems differing only in the composition of the gelator.

**Scheme 1:**
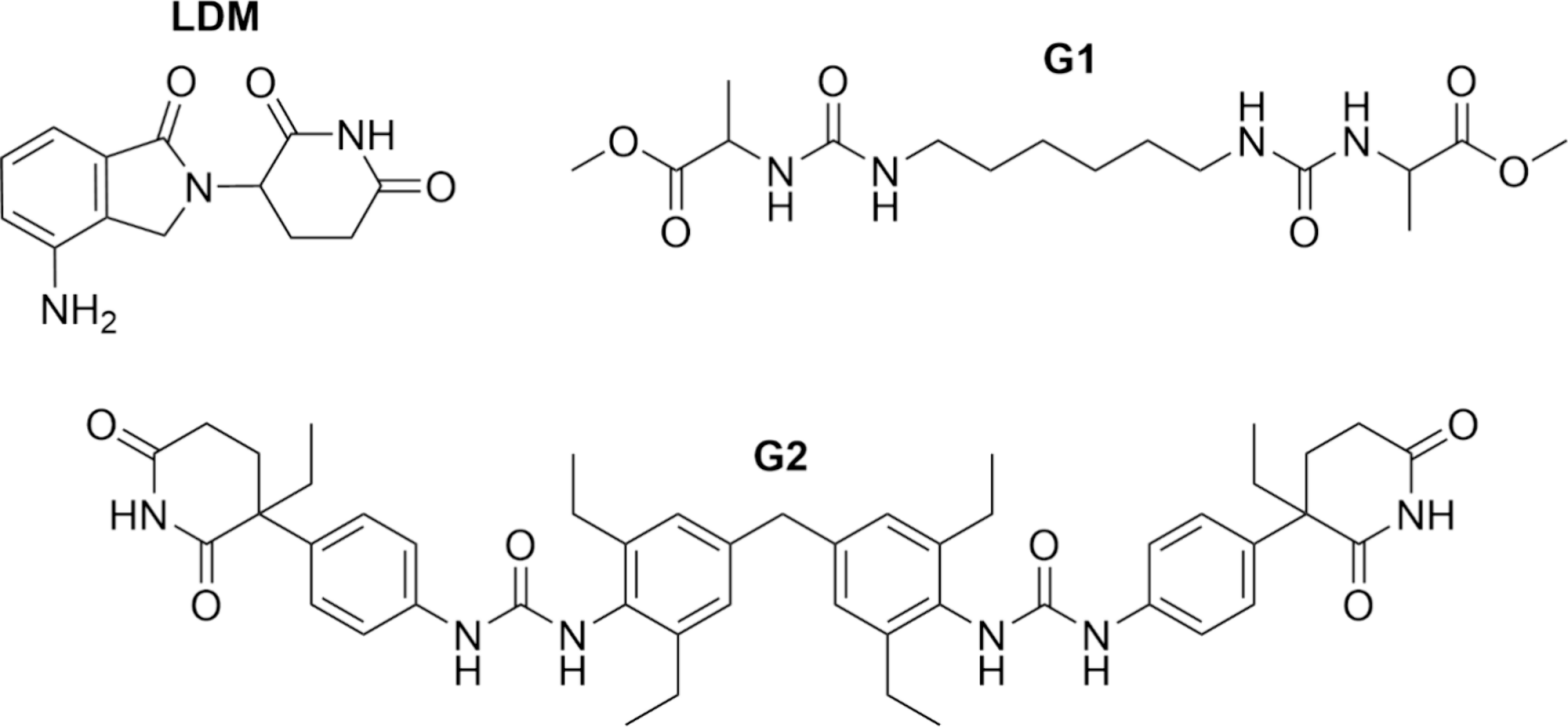
Chemical structures of Lenalidomide (LDM) and Gelators **G1** and **G2** ^a^

a The structure of **G1** is unrelated to LDM, whereas **G2** contains imide moieties in its end groups that mimic the imide moiety in LDM.

**Table 2:** Gelation Results in Good Solvents of LDM, as well as CGC and MGC (% w/v) for Gelators **G1** and **G2** ^a^

**solvent**	**G1**	**CGC**	**MGC**	**G2**	**CGC**	**MGC**
methanol	IS	-	-	IS	-	-
ethanol	G	0.7	1.0	IS	-	-
propanol	P	-	-	IS	-	-
1-butanol	P	-	-	IS	-	-
2-butanol	P	-	-	IS	-	-
cyclopentanone	G	0.5	2.0	G*	1.5	2.0
cyclohexanone	P	-	-	P	-	-
acetone	G	1.0	2.0	IS	-	-
acetonitrile	G	0.4	1.0	IS	-	-
nitrobenzene	G	0.2	2.0	G	0.1	2.0
nitromethane	IS	-	-	IS	-	-
1,4-dioxane	G	1.0	2.0	G	1.0	2.0
THF	G	0.5	1.0	P	-	-
methanol + DMSO	IS	-	-	G	2.0	2.5
ethanol + DMSO	P	-	-	P	-	-
propanol + DMSO	P	-	-	P	-	-
1-butanol + DMSO	P	-	-	G	1.5	1.8
2-butanol + DMSO	P	-	-	G	0.5	2.5

a IS: insoluble; P: precipitates on cooling; G: gel; G*: gels with sonication. Alcoholic solvents labelled “+DMSO” had a few drops of DMSO added to improve the dissolution of the gelator, as previous work has shown it to improve the solubility of **G2** and enable gelation of alcoholic solvents.^9^

Representative gels of **G1** and **G2** in cyclopentanone at both the CGC and MGC were characterized by oscillatory rheometry. Shear stress sweeps (Figure 1a,b) show that both gels have considerably larger storage and loss moduli at MGC compared to those at CGC, along with a higher yield stress, indicating the formation of stronger gels at higher gelator concentration. Frequency sweeps (Supporting Information Figure S1) corroborate these findings, showing that the gels have a constant viscosity over a range of frequencies when exposed to shear stress within the linear viscoelastic region (LVR). SEM images (Figure 1c–e) show the structure of xerogels produced by drying the cyclopentanone gels of **G1** and **G2**. The gels at their MGC generally comprise linear ribbon-like fibers approximately 500 nm in width, forming a physically entangled network. By contrast, the gel at CGC has a less well-defined fibrous network, explaining the difference in the mechanical strength.

**Figure 1:**
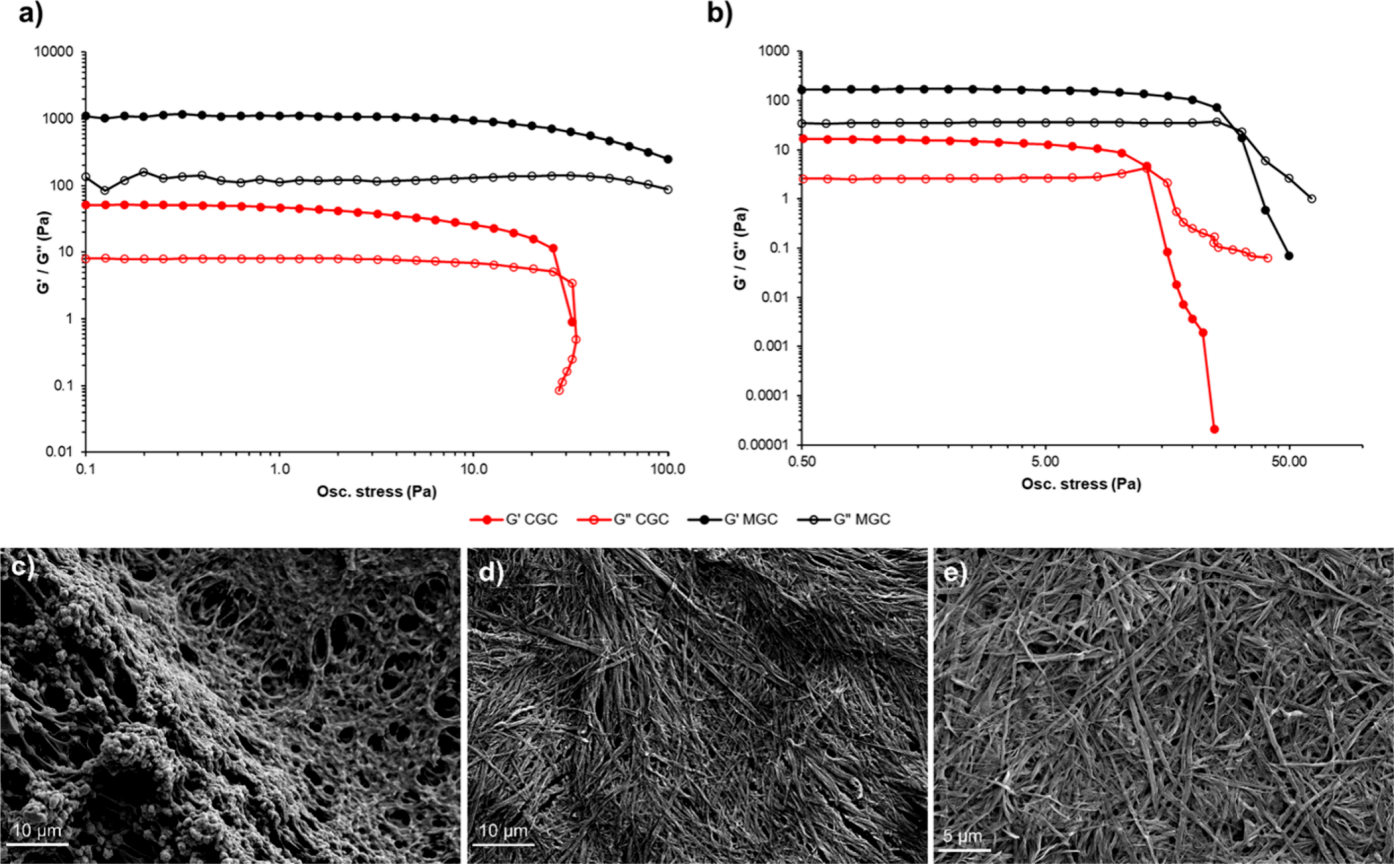
(a, b) Oscillatory stress–sweep rheological analysis of cyclopentanone gels of (a) **G1** and (b) **G2** at both CGC and MGC as representative examples, measured at a constant frequency of 1 Hz. Gels are characterized by a *G*′ that is approximately an order of magnitude greater than *G*″ up to the yield stress. Both gel systems have considerably larger storage and loss moduli at MGC compared to CGC. (c, d) SEM images of xerogels produced from gels of (c) **G1** cyclopentanone (CGC), (d) **G1** cyclopentanone (MGC), and (e) **G2** cyclopentanone (CGC).

### Gel-Phase Crystallization

Gel-phase recrystallization experiments were performed at a consistent supersaturation of LDM in each solvent at both the CGC and the MGC of the gelator separately. The crystals grown within gel media are difficult to characterize by optical microscopy due to the opacity of most **G1** and **G2** gels, but in general, they appear to grow at a similar rate to the solution-phase control experiments and were small, in some cases only visible by SEM (Figure 2). Not all experiments produced crystals of sufficient size and quality for unit cell determination by SC-XRD analysis, but all samples could be analyzed by XRPD. However, attempts to dissolve the **G1** and **G2** gels by addition of anions,^28,29,39^ sonication, stirring, or heating were unsuccessful, meaning that the crystals could only be transferred to XRPD slides by removing them with a spatula. As a result, in most cases, the powder patterns of the LDM crystals are convoluted with those of the dried **G1** or **G2** xerogels. A combination of XRPD and SC-XRD was used, where possible, to determine the polymorphic outcome of each experiment, and the results for gel-phase crystallization experiments at both CGC and MGC are shown alongside the solution-phase control experiments in Table 3. The presence of LDM crystals has varying effects on the strength of the resulting **G1** and **G2** gels, as observed by rheometry (Supporting Information Figure S3). While the presence of crystals appears to weaken gels of **G1** and **G2** in cyclopentanone at their CGC and MGC, respectively, with decreased storage and loss moduli and a lower yield stress, the **G1** cyclopentanone gel at MGC is relatively unaffected, and the **G2** cyclopentanone gel at CGC is stronger and higher yielding. If the drug crystals grow before or at the same time as the gel fibers, this indicates that depending on the gel system, the presence of crystals may either inhibit the growth and entanglement of gel fibers to form a strong three-dimensional network or instead make the fibrous network stronger and more rigid, perhaps through inclusion of small crystalline particles within the gel fibers themselves. Alternatively, if the gel network forms before any drug crystals grow, it suggests that interactions between the growing fibers and the drug in solution have an effect on the resulting fibrous network. Qualitative assessment via a vial inversion test indicates that the gels form within several minutes of sonication in most cases; however, due to the opacity of the resulting samples, it is unclear how rapidly the LDM crystals grow within the gel media.

**Figure 2:**
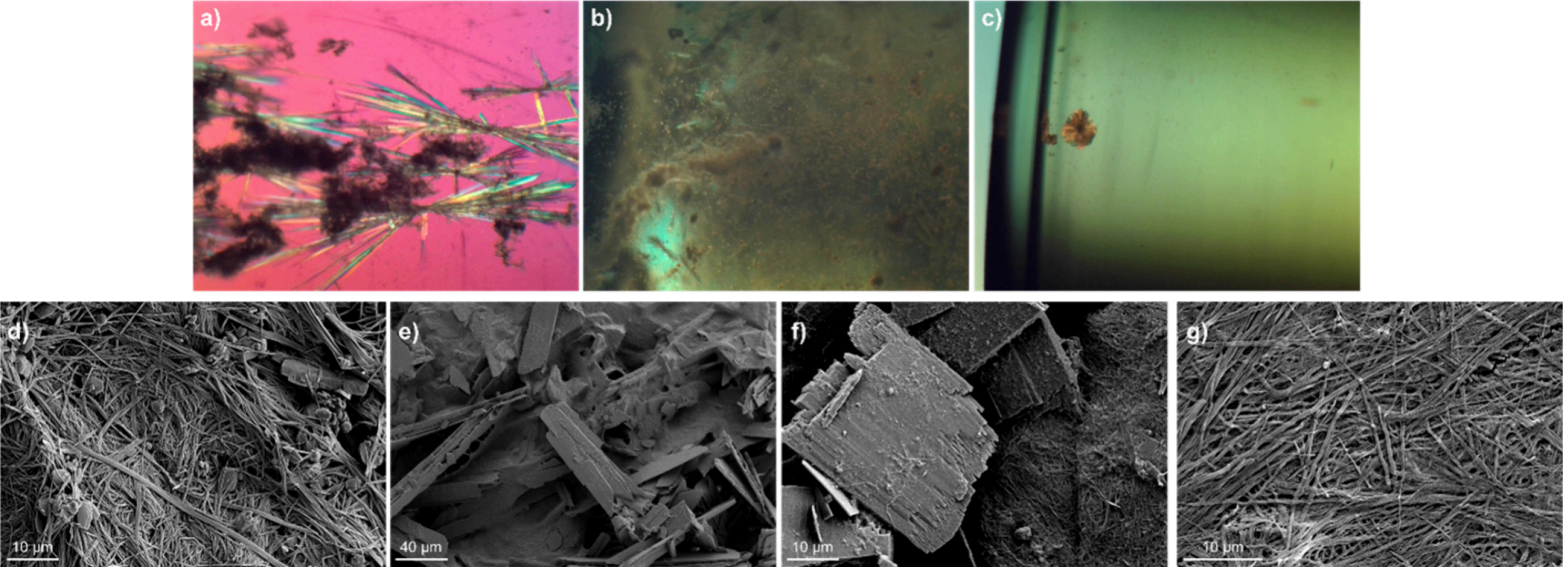
Polarized optical microscopy images of LDM crystals grown inside gels of (a) **G1** ethanol, (b) **G1** cyclopentanone, and (c) **G2** nitrobenzene all at CGC. SEM images of crystals grown inside gels of (d) **G1** dioxane, (e) **G1** cyclopentanone (CGC), (f) **G1** cyclopentanone (MGC), and (g) **G2** cyclopentanone (MGC). LDM crystals grown within gel media were typically of insufficient size and quality for unit cell determination by SC-XRD analysis, with SEM images showing crystals of approximately 20–150 μm in length depending on the xerogel sample.

**Table 3:** Polymorphic Outcome from Gel-Phase LDM Recrystallization Experiments, Determined from a Combination of XRPD and SC-XRD Analysis^a^

	**solvent**	**crystallization outcome**		
**gelator**	**solvent**	**CGC**	**MGC**	**control**
1	EtOH	Form 4 + **G1**	Form 4 + **G1**	Form 1
1	dioxane	Form 1	Form 1	Forms 1 + 2
1	acetone	**G1**	**G1**	Form 6
1	MeCN	**G1**	**G1**	Forms 1 + 2
1	nitrobenzene	Form 4 + **G1**	amorphous	Form 4
1	cyclopentanone	Form 8 + **G1**	Form 1 + **G1**	Form 1
1	THF	Form 1 + **G1** Forms A + B	Form 1 + **G1**	Form 1
2	MeOH+DMSO	amorphous	amorphous	Form 4
2	dioxane	Form 2 + **G2**	Form 2 + **G2**	Forms 1 + 2
2	nitrobenzene	amorphous	**G2**	Form 4
2	cyclopentanone	Form 1	Form 1	Form 1
2	1-BuOH+DMSO	amorphous	amorphous	Forms 1 + 5
2	2-BuOH+DMSO	Form 5	**G2**	Form 5

a LDM Form 8 refers to a novel cyclopentanone hemi-solvate discovered by SC-XRD analysis of the gel-crystal mixture. Unless otherwise specified, **G1** crystallized in the previously reported Form A structure.^39^

While many gel-phase recrystallization experiments using **G1** produce the same polymorphic outcome as the control experiments (Supporting Information Figure S4), LDM crystallization within gels of **G1** in three solvents differs from the control experiments in the absence of gelator (Supporting Information Figure S5). XRPD and SC-XRD analyses show that while the control crystallization of LDM from ethanol produces a pure phase of Form 1, **G1** gels reproducibly contain crystals of the metastable anhydrous Form 4 phase instead, alongside crystals of the **G1** gelator (Figure 3a). This indicates that the metastable Form 4 can be grown directly from the gel phase as opposed to the previously reported methods of dehydrating Forms 2 or 7,^33^ offering a direct route to crystallizing this polymorph. XRPD analysis of the **G1** dioxane gel-phase recrystallization experiment at CGC revealed a unique powder pattern, similar to but distinct from the **G1** dioxane control gel containing no LDM, unlike the same experiment at MGC, which matches the control gel (Figure 3b). SC-XRD analysis on both samples revealed crystals of LDM Form 1 but not LDM Form 2, which both grow concomitantly from the control crystallization in the solution phase. While this may be evidence that the **G1** gels of dioxane could prevent the concomitant crystallization of LDM Forms 1 and 2 and produce only Form 1, the presence of Form 2 within the gel cannot be ruled out.

**Figure 3:**
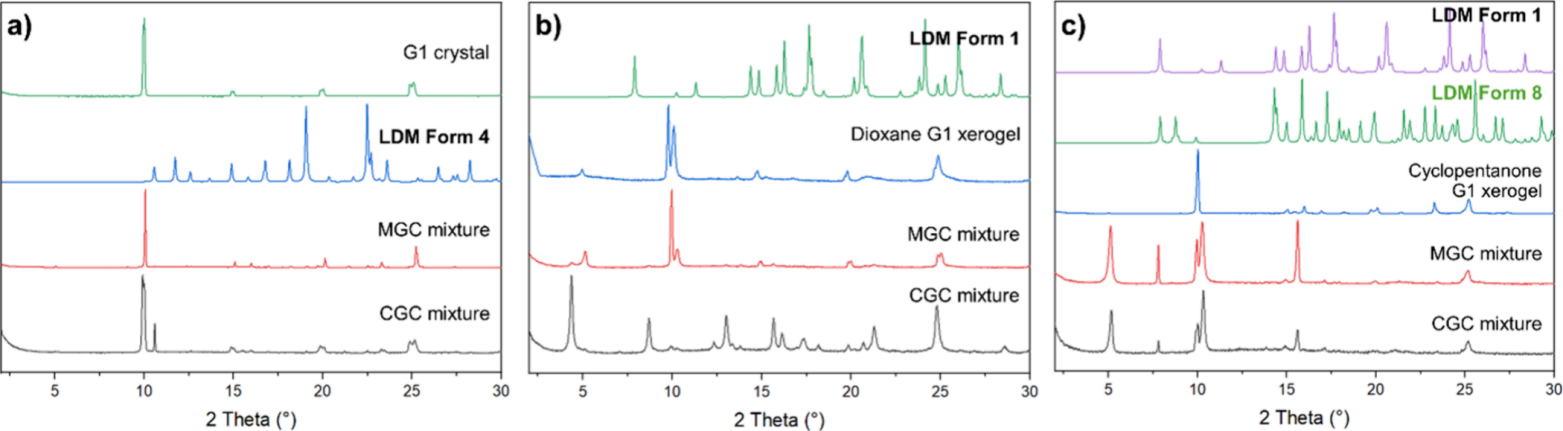
XRPD patterns of gel-crystal mixtures analyzed from LDM recrystallization experiments in **G1** gels of (a) ethanol, (b) dioxane, and (c) cyclopentanone. Gel-crystal mixtures obtained from experiments at both CGC and MGC are compared. In panels (b) and (c), a control gel of **G1** containing no LDM was also analyzed as a dried xerogel for comparison. This data was used in combination with single-crystal XRD to determine the polymorphic outcome from crystallization within **G1** gels. The powder pattern for LDM polymorphs and solvates, including Form 8 (cyclopentanone hemisolvate), was simulated from the SC-XRD data and shown in bold. Overlay plots comparing the experimental gel-crystal mixture patterns and simulated LDM crystal patterns are presented in Supporting Information Figure S5.

Most notably, crystallization within **G1** gels of cyclopentanone affords crystals of a novel cyclopentanone hemisolvate of LDM (referred to henceforth as LDM Form 8), and the structure was determined by SC-XRD (Figure 4). Form 8 contains 1.0 mol of LDM and 0.5 mol of cyclopentanone, with two molecules of LDM and one molecule of cyclopentanone in the asymmetric unit. One molecule of each LDM enantiomer is present in the asymmetric unit, with each enantiomer involved in a different intermolecular hydrogen-bonding arrangement. Separate enantiomers make homodimer interactions via $R_{2}^{2}$ ­(10) hydrogen-bonded ring motifs^45^ consisting of two N103–H103···O103 or N203–H203···O202 interactions for the *R*- and *S*- enantiomers, respectively, with N···O distances of 2.919(3) and 2.826(3) Å, respectively. The R-enantiomers also form head-to-tail interactions along the *b* axis via N102–H10A···O103 interactions. *R*- and *S*-enantiomers interact with each other via N202–H20A···O101 interactions with an N202···O101 distance of 2.976(4) Å. The LDM *S*-enantiomers interact with cyclopentanone molecules via N202–H20B···O1 interactions with an N202···O1 distance of 3.058(8) Å. The XRPD pattern (Figure 3c) of the gel-crystal mixture sampled from the experiment is dominated by the xerogel peaks and a pure phase could not be obtained by dissolving the gel. Attempts to reproduce this crystal by cooling crystallization or slurry of LDM (starting as Form 1 or 2) in cyclopentanone in the absence of gel for 2 weeks were unsuccessful, yielding only LDM Form 1 (Supporting Information Figure S6). This may indicate that LDM Form 8 can be obtained only by gel-phase crystallization.

**Figure 4:**
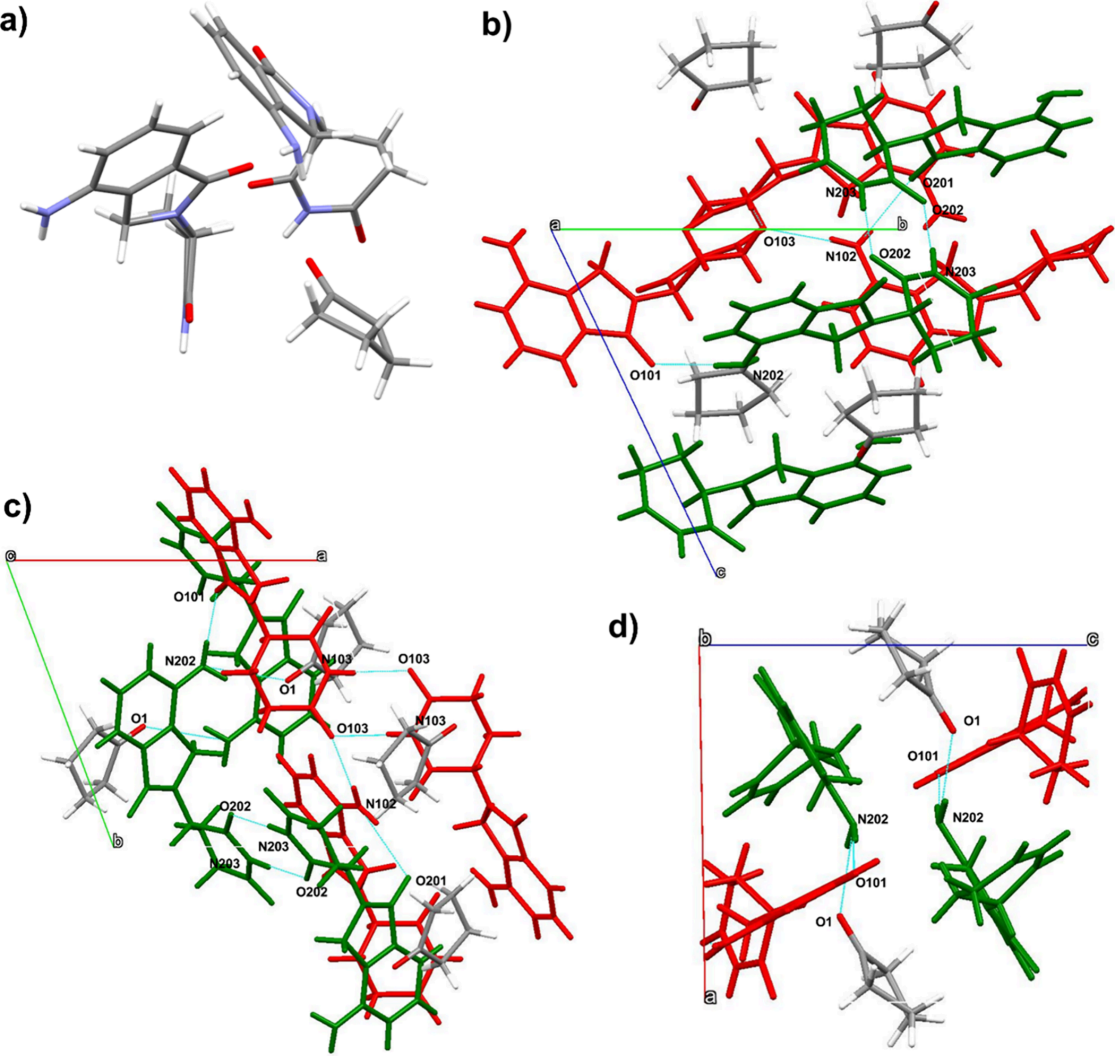
Crystal structure of LDM Form 8 (cyclopentanone hemisolvate). Views of (a) the asymmetric unit containing two enantiomers of LDM and one molecule of cyclopentanone and (b–d) views down unit cell axes, with LDM enantiomers *R* and *S* shown in green and red, respectively. Each LDM enantiomer makes homodimer interactions via $R_{2}^{2}$ ­(10) hydrogen-bonded ring motifs consisting of two N103–H103···O103 or N203–H203···O202 interactions for the *R*- and *S*-enantiomers respectively, with N···O distances of 2.919(3) Å and 2.826(3) Å, respectively. *R*- and *S*- enantiomers interact with each other via N202–H20A···O101 interactions with an N202···O101 distance of 2.976(4) Å. The LDM *S*-enantiomers interact with cyclopentanone molecules via N202–H20B···O1 interactions with an N202···O1 distance of 3.058(8) Å.

A new polymorph of the gelator **G1** (referred to henceforth as **G1** Form B) was also discovered upon analysis of the gel-phase recrystallization experiments from the **G1** tetrahydrofuran gels, in which LDM Form 1 grows alongside gelator crystals at both CGC and MGC. The structure of **G1** Form B was solved via single-crystal X-ray diffraction (Figure 5a–c), revealing similar features to the originally reported Form A, such as the $R_{2}^{1}$ ­(6) urea α-tape hydrogen-bonding motif.^39^ The powder pattern of Form B simulated from the SC-XRD data is very similar to that of Form A, obtained directly from the synthesis (Figure 5d).

**Figure 5:**
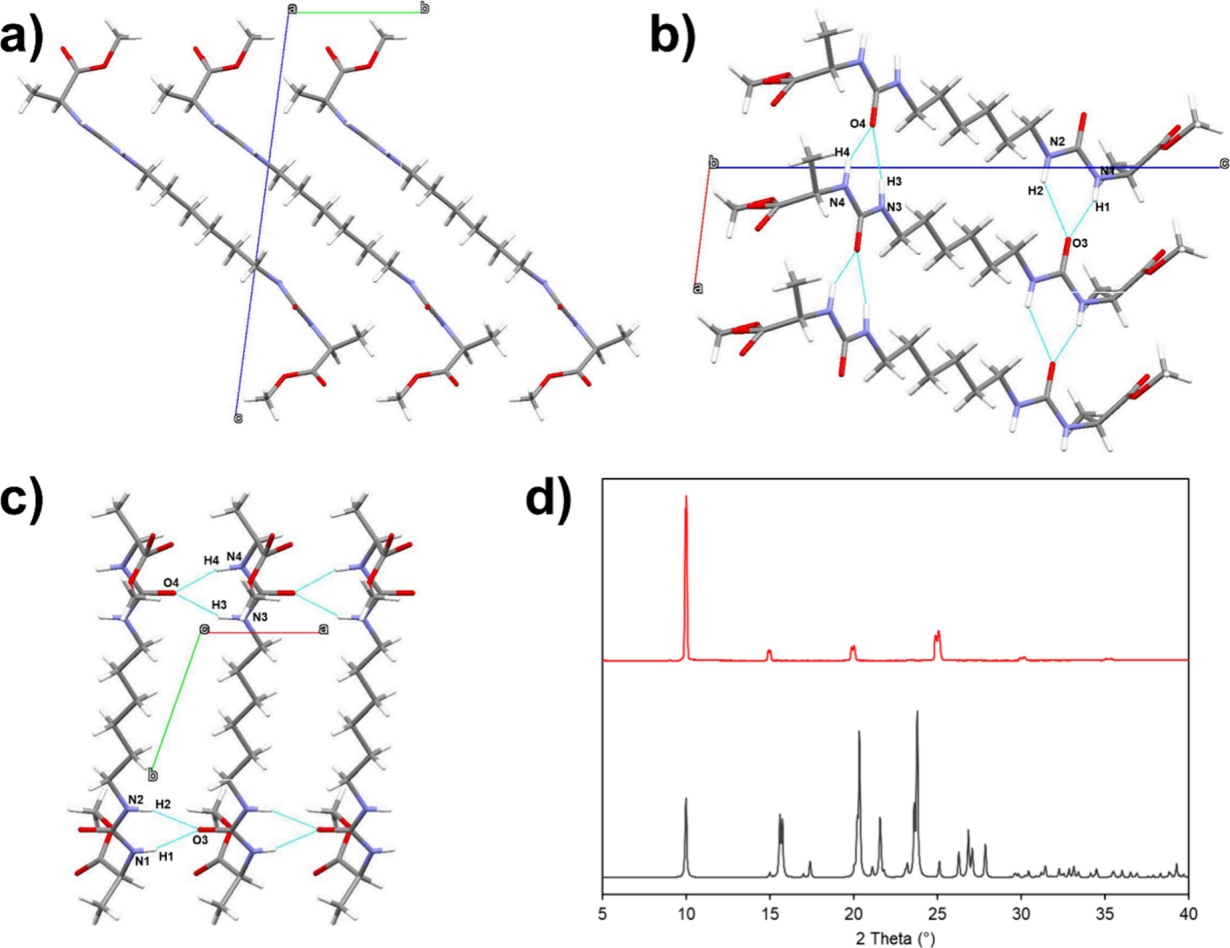
(a–c) Views down the unit cell axes of the crystal structure of **G1** Form B. Similarly to Form A,^39^ the **G1** molecules stack via a pair of $R_{2}^{1}$ ­(6) urea α-tape hydrogen-bonding motifs via N1–H1···O3, N2–H2···O3, N4–H4···O4, and N3–H3···O4 interactions. (d) XRPD diffractograms of **G1** Form A obtained from synthesis (top) compared to Form B powder pattern simulated from SCXRD data (bottom).

By contrast, the results in Table 3 show that crystallization within **G2** gels does not change the polymorphic outcome compared with the solution-phase control experiments in all solvents except dioxane. As for the **G1** dioxane gels, the gel-phase crystallization experiments in **G2** dioxane gels at both CGC and MGC produce a different XRPD pattern compared to the **G2** dioxane control gel containing no LDM (Supporting Information Figure S7), and unit cell determination by SC-XRD analysis finds only LDM Form 2, unlike the analogous **G1** gel-phase crystallization experiments, where only Form 1 is found. However, the XRPD patterns cannot confirm the phase purity of the Form 2 crystals identified by SC-XRD due to the broad and intense xerogel peaks; therefore, concomitant crystallization of Form 1 cannot be ruled out as for the analogous **G1** case. The challenge in analyzing the polymorphic outcome of these samples is compounded by the generally poor crystallinity of LDM grown within the **G2** gels, with some samples found to be completely amorphous by XRPD. Crystallization experiments in **G2** cyclopentanone gels produce only Form 1, with no amount of the novel Form 8 detected. These results contrast to previously reported examples where the drug-mimetic gelator exerted control over the polymorphic outcome of the crystallizing API, whereas nonmimetic gelators had no such effect.^8,9,11^ In the present case, the nonmimetic **G1** gelator has been used to discover a new LDM solvate and is capable of growing the metastable anhydrous form rather than the thermodynamic form in certain solvent systems, while the drug-mimetic gelator has not. This is particularly interesting since the same **G2** gelator was previously shown to be capable of affecting the crystallization behavior of a very similar API thalidomide.

Gel-phase crystallization experiments were also conducted in gel blends of **G1** and **G2** in varying molar ratios in both cyclopentanone and dioxane. Given the considerable structural differences between the **G1** and **G2** molecules, in particular the difference in linker, it is likely that the blended gels consist of two independent, noninteracting fibrous networks where **G1** and **G2** fibers grow orthogonally,^39^ although it is possible that they entangle with each other to form a 3-dimensional network. XRPD analysis of the resulting gel-crystal mixtures (Supporting Information Figure S8) is complicated by the presence of strong gelator signals and poor crystallinity of the LDM crystals, but in cyclopentanone, it appears that the novel LDM Form 8 can grow from gels composed of any composition containing at least 20% w/w of **G1** (Supporting Information Table S6). This suggests that simply the presence of some **G1** fibers within the gel network is sufficient to induce the growth of LDM Form 8, and the presence of **G2** has a neutral effect. XRPD analysis of the dioxane gels shows that xerogel signals dominate the diffractogram at all gelator ratios, and the LDM is poorly crystalline, although it appears that Form 2 may crystallize at higher ratios of **G1**. Since the pure **G1** gels previously analyzed appeared to contain only Form 1 instead of the concomitant mixture, this evidence suggests that these gels probably do not prevent the concomitant crystallization of Forms 1 and 2.

### Drug–Gelator Interactions

It is unclear whether interactions between drug and gelator are responsible for the previously reported examples of supramolecular gelators controlling the API crystallization outcome. If the drug and gel fibers interact strongly, these interactions may be detectable when the drug and gelator are in solution. Suitable methods to study these interactions are titration and diffusiometry. Solutions of **G1** and **G2** at 1.4 mg/mL (below CGC) in dioxane-*d*_8_ contain gelatinous precipitates at room temperature, which can be dissolved to produce clear solutions suitable for solution-state NMR analysis at 90 °C. Dioxane-*d*_8_ solutions of LDM were titrated into these hot gelator solutions to study potential drug–gelator interactions in the resulting solution, increasing from a 25-fold excess of gelator to a 4.5-fold excess of drug (Figure 6). In both samples, the LDM integrals increase concomitantly with the addition of LDM. In the **G1** sample (Figure 6a), very few signals corresponding to either drug or gelator change in chemical shift with increasing LDM concentration, aside from a small increase of 0.02–0.03 ppm in the **G1** urea-NH signals at approximately 5.0 and 5.1 ppm and an increase of 0.05 ppm in the LDM NH signals at 9.5 and 10.4 ppm corresponding to the amine and imide groups, respectively. These NH signals are particularly sensitive to water content, NH-water exchange, inter- and intramolecular interactions, and viscosity. Because of this, changes in only the NH signals and no other signals are insufficient evidence of strong intermolecular interactions between LDM and **G1** or self-association of either component. In the **G2** sample (Figure 6b), there are more signals that change chemical shift upon addition of LDM, including several **G2** aromatic-region signals from both the linker and the end groups, and the NH moieties in **G2** (urea and imide), all increasing in chemical shift by around 0.05–0.07 ppm. This suggests that intermolecular interactions involving **G2** are more likely, with several regions of the molecule displaying changes in the chemical shift. However, many neighboring signals show no detectable variation, and the only LDM signals showing any shift are again the NH signals, which increase in chemical shift by approximately 0.07 ppm. Given the magnitude of the changes observed, strong drug–gelator interactions are unlikely in either system. This is supported by NMR diffusiometry studies (diffusion-ordered spectroscopy, DOSY) conducted in DMSO-*d*_6_ (Supporting Information entry Figure S9). Strong interactions between either **G1** or **G2** and LDM in solution would result in both molecules having the same (or close) diffusion coefficients, but this was not the case.

**Figure 6:**
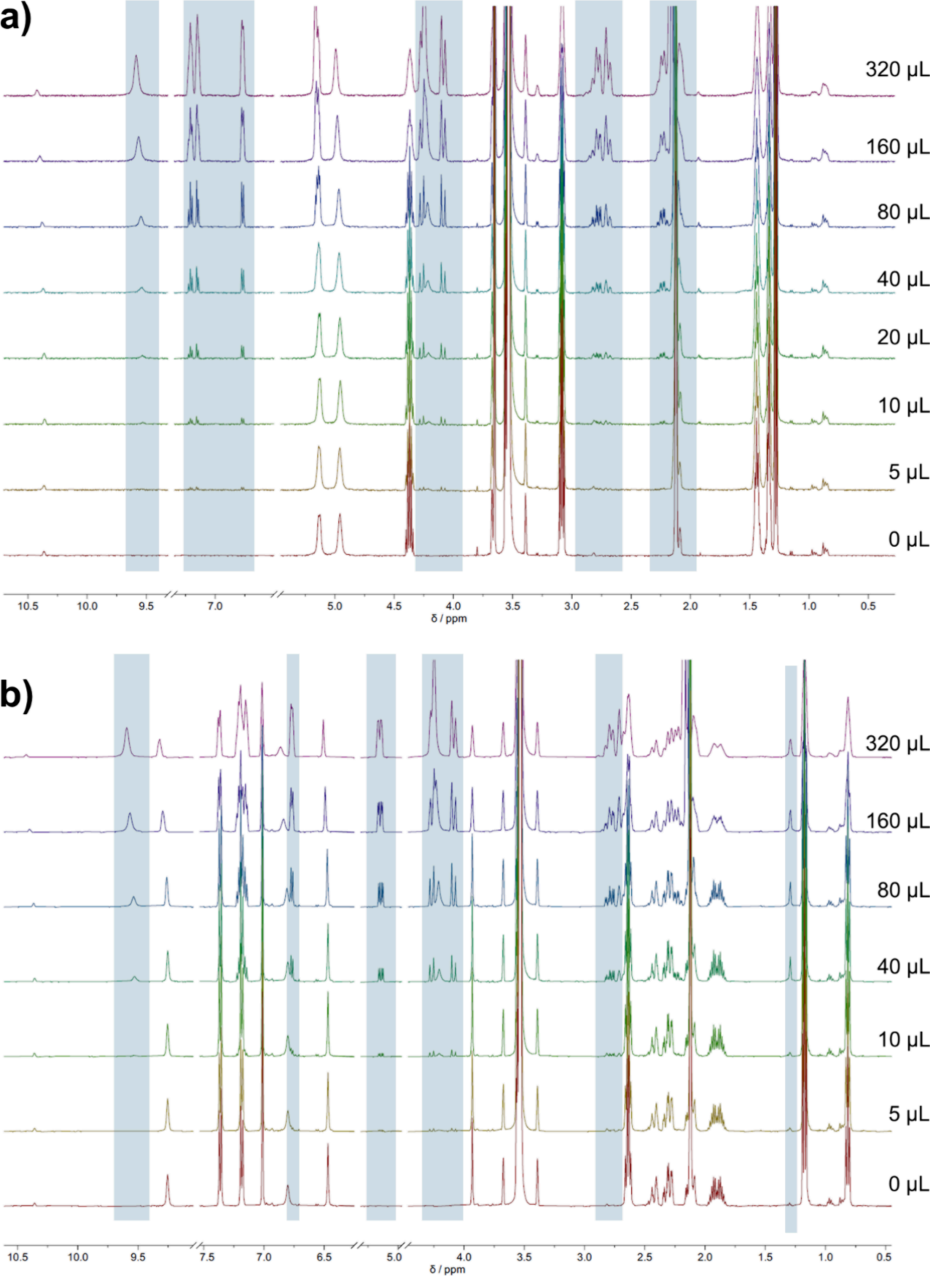
500 MHz ^1^H NMR titration plots for dioxane-*d*_8_ gels of (a) **G1** and (b) **G2** (initially at 1.4% w/v) as aliquots of a dioxane-*d*_8_ solution of LDM (7 mg/mL) were added. Residual solvent peaks for dioxane and water are present as strong signals at 3.5 and 2.1 ppm, respectively. The gelator–drug molar ratio increased from 1:0.04 up to 4.5:1 over eight steps. LDM signals increase in intensity as larger aliquots are added, but there are no significant changes in chemical shift for either the drug or gelator component in either titration, suggesting that no strong binding interactions are present in the solution state.

Although no strong interactions were detected, NMR titrations in solution do not reveal whether LDM molecules could become physically incorporated in the gel fibers when they form, potentially affecting the surface composition of the fibers, which may link to the observed crystallization phenomena. However, the ^1^H NMR spectra of a **G1** gel of dioxane-*d*_8_ at 1.5% w/v containing a fixed 0.5% w/v of LDM obtained at 25, 50, and 90 °C (Supporting Information Figure S10) also show that as the gelatinous sample is warmed up and dissolves, the signals corresponding to **G1** increase in intensity while the LDM signals do not change in integral relative to the residual DMSO-*d*_6_ solvent peak, indicating that no significant quantity of LDM molecules are incorporated into the **G1** gel fibers. This may differ in the **G1** gels of cyclopentanone and/or ethanol and therefore be linked to the observed differences in crystallization outcome compared to the solution phase controls; however, these solvents were intractable for solution state NMR studies.

Taken together, this suggests that if any drug–gelator interactions linked to the gel-phase crystallization phenomena are present, they are too weak to detect by NMR in the solution state. This means that the observed crystallization phenomena are likely related to general gel-phase properties such as limited molecular diffusion and/or convection, the removal of active surfaces from glass or dust to provide a homogeneous medium for nucleation, or the presence of the gel fibers as an active surface with a periodicity that can transfer to the growing API crystal through heterogeneous secondary nucleation.^10^ If this is the case, then the periodicity of the **G1** fibers may be such that it can template the growth of LDM Form 2 in ethanol and LDM Form 8 in cyclopentanone, while the periodicity of the **G2** fibers does not. This is also compatible with the observation that LDM Form 8 could be grown within blended gels at a **G1** ratio as low as 20% w/w, assuming that **G1** and **G2** fibers grow orthogonally and that the presence of some **G1** fibers templates the growth of Form 8 while the remaining **G2** fibers are inert with respect to crystal growth. Alternatively, a recent discovery is that confinement can have a significant effect on polymorph crystallization.^46–48^ For example, crystallization from a structured ternary fluid can influence polymorph nucleation by restricted diffusion locally perturbing supersaturation levels and influencing the free energy barrier for the growth of different polymorphs.^48^ While the mesh size of the **G1** and **G2** gels are unknown, it is possible that they are on a comparable scale to the nanosized domains within structured ternary fluids, and the confinement effect provided by **G1** fibrous meshes influences the crystallization outcome.

## Conclusions

Crystallizing lenalidomide within gels formed with a nonmimetic bis­(urea) gelator revealed a novel hemisolvate of cyclopentanone and facilitated the direct growth of the metastable anhydrous form over the thermodynamic form in ethanol, offering an alternative route to dehydration of lenalidomide hydrates to obtain this metastable form. This demonstrates the usefulness of the gel-phase crystallization approach in pharmaceutical solid form screening. Crystallizing lenalidomide within equivalent gels of a drug-mimetic bis­(urea) gelator, however, demonstrated no such control over the polymorphic outcome nor produced the novel hemisolvate. Crystallization experiments in cogels of both gelators in cyclopentanone produced the cyclopentanone hemisolvate at all molar ratios except pure drug-mimetic gelator, suggesting that its growth is enabled simply by the presence of fibers of the nonmimetic gelator, and that by comparison, the drug-mimetic gel fibers have no effect on the crystallization outcome. Diffusiometry and NMR studies showed the interaction between lenalidomide and either gelator was not strong, suggesting that the mechanism by which the nonmimetic gelator affects crystallization is either via heterogeneous secondary nucleation and/or the periodicity of the gel fibers that it forms or via confinement effects and not through specific intermolecular interactions with the drug.

## Supplementary Material



## Data Availability

The underlying data files are available free of charge at DOI: 10.15128/r2tq57nr080. The crystal structures of LDM Form 8 and **G1** Form B described in this paper have been deposited in the CSD with CCDC reference codes 2453375 and 2453376, respectively.

## Notes

Synthetic procedures and characterization, lenalidomide solubility data, additional crystallographic data for lenalidomide Form 8 and **G1** Form B, oscillatory rheometry data, additional XRPD patterns, DOSY plots and NMR spectra collected at different temperatures (PDF)

## Abbreviations

API: active pharmaceutical ingredient
LMWG: low-molecular-weight gelator
SC-XRD: single-crystal X-ray diffraction
LDM: lenalidomide
XRPD: X-ray powder diffraction
NMR: nuclear magnetic resonance
ES: electrospray
CGC: critical gelation concentration
MGC: maximum gelation concentration
SEM: scanning electron microscopy
DOSY: diffusion-ordered NMR spectroscopy
