# Supplementary material for: Nonmimetic Gels Direct Novel Crystallization Behavior of Lenalidomide
Source: Cryst Growth Des. 2025 Sep 25;25(20):8646–57. doi: 10.1021/acs.cgd.5c01083 (PMC12532200; doi:10.1021/acs.cgd.5c01083)
Supplement: Supplementary file 1 [file cg5c01083_si_001.pdf]

SUPPLEMENTARY INFORMATION

Non-Mimetic Gels Direct Novel Crystallization Behavior of Lenalidomide

Martin A. Screen,[1] Juan A. Aguilar-Malavia,[1] Toby J. Blundell,[1] James F. McCabe,[2] Sean Askin,[3] Clare S. Mahon,[1] Mark R. Wilson,[1] Jonathan W. Steed[1,*]

[1]Durham University, Department of Chemistry, South Road, Durham DH1 3LE, United Kingdom; [2]Early Pharmaceutical Development & Manufacture, Pharmaceutical Sciences, R&D, AstraZeneca, Macclesfield SK10 2NA, United Kingdom; [3]Advanced Drug Delivery, Pharmaceutical Sciences, R&D, AstraZeneca, Cambridge CB2 0AA, United Kingdom. *Tel: +44 191 334 2085; Email: jon.steed@durham.ac.uk

Gelator G1 synthesis and characterization. L-alanine methyl ester hydrochloride (1.594 g, 11.42 mmol) was dissolved in chloroform (40 mL) and triethylamine (1600 µL, 11.47 mmol) was added. 1,6-Diisocyanatohexane (910 µL, 5.67 mmol) was added dropwise then the solution was heated under reflux. After 1 h, the solution was cooled to room temperature and the gelatinous precipitate was filtered and washed with dichloromethane (2×10 mL) and then diethyl ether (10 mL). The resulting solid was sonicated in warm water (40 °C) for 30 min then filtered, recrystallized from methanol, filtered again and washed with diethyl ether. The compound was dried in a drying pistol for 6 h and the product was obtained as a white powder (1.074 g, 2.400 mmol, 42 % yield). ^{1}H NMR (400.20 MHz, DMSO-d_6): δ=6.16 (2 H, d, J=7.7 Hz, N(10,19)H), 5.93 (2 H, t, J=5.6 Hz, N(5,8)H), 4.11 (2 H, p, J=7.3 Hz, NHC(12,21)H), 3.58 (6 H, s, OC(16,26)H_3), 2.99–2.86 (4 H, m, NHC(1,7)H_2), 1.30 (6 H, d, J=6.4 Hz, C(13,22)H_3), 1.23–1.17 ppm (8 H, m, C(2,3,4,6)H_2). ^{13}C{^{1}H}-NMR (100.63 MHz, DMSO-d_6): δ=174.75 (COO), 157.79 (NCO), 52.09 (OCH$_3$), 48.55 (NHCHCH$_3$), 40.7–39.7 (NCH$_2$CH$_2$ under DMSO residual solvent peak), 30.36 (NCH$_2$$CH_2$), 26.51 (CH$_2CH_2$$CH_2$), 18.45 ppm (CH$CH_3$). m/z (ES^{+}-MS): 375 ([M+H]$^{+}$, 79 %), 397 ([M+Na]$^{+}$, 100 %), 771 ([2 M+Na]$^{+}$, 10 %). Elemental analysis calculated (%) for $C_{16}H_{30}N_4O_6$: C 51.32, H 8.08, N 14.96; found: C 50.59, H 8.06, N 14.84.

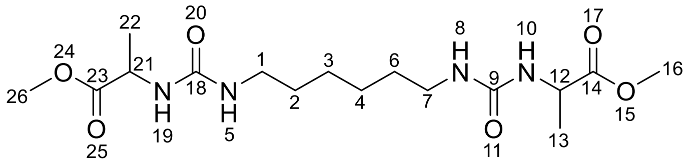

Gelator G2 synthesis and characterization. DL-aminoglutethimide (0.7392 g, 3.186 mmol) was dissolved in chloroform (45 mL) and an excess of triethylamine (0.6 mL) added dropwise with continuous stirring. A solution of 4,4′-methylenebis(2,6-diethylphenyl isocyanate) (0.6150 g, 1.699 mmol) in chloroform (30 mL) was added dropwise and the reaction mixture stirred at 70 °C for 24 h. The resulting white precipitate was isolated by filtration, washed with chloroform (2×15 mL) and dried in a drying pistol for 4 h. The product was obtained as a white powder (0.4662 g, 0.5642 mmol, 35% yield). ^{1}H NMR (400.20 MHz, DMSO-d_6): δ=10.82 (2H, s, N(47,50)H), 8.80 (2H, s, NH), 7.55 (2H, s, NH), 7.45 (4H, d, J=8.82 Hz, H-Ph), 7.16 (4H, d, J=8.7 Hz, H-Ph), 7.00 (4H, s, C(4,6,9,13)H), 3.86 (2H, s, C(7)H_2), 2.59-2.38 (10H, m, CH_2),

2.36-2.26 (2H, m, CH_2), 2.23-2.10 (4H, m, CH_2), 1.82 (4H, dtt, J=21.1, 14.3, 7.3 Hz, CH_3-CH_2), 1.11 (12H, t, J=7.5 Hz, Ph-CH_2-CH_3), 0.76 (6H, t, J=7.4 Hz, CH_2-CH_3). $^{13}C\{^1H\}$-NMR (100.63 MHz, DMSO-d_6): δ=176.3, 173.2, 154.3, 142.3, 140.1, 139.7, 132.6, 132.2, 127.0, 126.7, 118.2, 50.0, 32.6, 29.5, 26.4, 24.9, 15.1, 9.3 ppm. m/z (ES^+-MS): 827 ($[M+H]^+$, 20 %), 849 ($[M+Na]^+$, 100 %). Elemental analysis calculated (%) for $C_{49}H_{58}N_6O_6$: C 71.16, H 7.07, N 10.16; found: C 70.31, H 6.80, N 9.97.

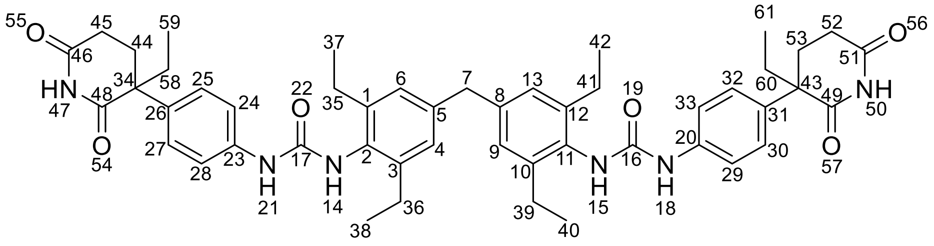

Table S1. Solubilities of LDM in a range of solvents at room temperature, determined via the gravimetric method.

Solvent	Solubility (mg/mL)	Solvent	Solubility (mg/mL)	Solvent	Solubility (mg/mL)
Chloroform	0.4	Cyclohexane	0.0	1-butanol	1.4
Toluene	0.0	p-xylene	2.0	2-butanol	1.4
Tetrahydrofuran	4.2	Nitromethane	5.4	1-propanol	0.4
Acetone	1.8	Nitrobenzene	1.2	1,4-butanediol	0.0
Dichloromethane	0.8	Cyclohexanone	8.9	Ethanol	1.7
DMSO-H_2O (1:1)	8.8	Cyclopentanone	9.4	Methanol	3.1
MeOH-H_2O (1:1)	3.0	Pentanol	0.2	Acetonitrile	3.1
THF-H_2O (1:1)	7.6	1,4-dioxane	7.8	Ethyl acetate	2.1

Table S2. Crystallographic data for LDM Form 8 (cyclopentanone hemi-solvate).

**Empirical formula**	$C_{15.5}H_{17}N_3O_{3.5}$	**μ / mm^{-1}**	0.104
**Formula weight**	301.32	**F(000)**	636.0
**Temperature / K**	120.00	**Crystal size / mm**	0.076 × 0.049 × 0.025
**Crystal system**	triclinic	**Radiation**	Mo Kα (λ = 0.71073)
**Space group**	$P\bar{1}$	**2Θ range for data collection / °**	3.968 to 51.398
**a / Å**	11.1851(7)	**Index ranges**	-13 ≤ h ≤ 13, -14 ≤ k ≤ 14, -15 ≤ l ≤ 15
**b / Å**	12.1666(8)	**Reflections collected**	38307
**c / Å**	12.6313(8)	**Independent reflections**	5315 [R_{int} = 0.1340, R_{sigma} = 0.0937]
**α / °**	62.187(2)	**Data/ restraints/ parameters**	5315/0/421

**β / °**	78.555(2)	**Goodness-of-fit on F^2**	1.006
**γ / °**	66.633(2)	**Final R indexes [I >=2σ (I)]**	R_1 = 0.0574, wR_2 = 0.1020
**Volume / Å³**	1395.46(16)	**Final R indexes (all data)**	R_1 = 0.1231, wR_2 = 0.1243
**Z**	4	**Largest diff. peak /hole**	0.45/-0.24
**ρ_{calc} g/cm³**	1.434		

Table S3. Hydrogen bonds for LDM Form 8 (cyclopentanone hemi-solvate).

D	H	A	d(D-H)/Å	d(H-A)/Å	d(D-A)/Å	D-H-A/°
N103	H103	O103[1]	0.87(3)	2.05(3)	2.919(3)	174(3)
N202	H20A	O101	0.90(4)	2.07(4)	2.976(4)	176(3)
N203	H203	O202[2]	0.90(3)	1.93(3)	2.826(3)	174(3)

[1]2-X,1-Y,-Z; [2]1-X,2-Y,-Z

Table S4. Crystallographic data for **G1** Form B.

**Empirical formula**	$C_{16}H_{30}N_4O_6$	**μ / mm⁻¹**	0.101
**Formula weight**	374.44	**F(000)**	202.0
**Temperature/K**	120	**Crystal size / mm**	0.191 × 0.077 × 0.019
**Crystal system**	triclinic	**Radiation**	Mo Kα (λ = 0.71073)
**Space group**	*P*1	**2Θ range for data collection/°**	4.6 to 54.712
**a / Å**	4.6439(4)	**Index ranges**	-5 ≤ h ≤ 5, -7 ≤ k ≤ 7, -22 ≤ l ≤ 23
**b / Å**	6.0527(5)	**Reflections collected**	13159
**c / Å**	17.9322(15)	**Independent reflections**	4128 [R_{int} = 0.0399, R_{sigma} = 0.0469]
**α / °**	95.476(3)	**Data/ restraints/ parameters**	4128/178/264
**β / °**	94.920(3)	**Goodness-of-fit on F^2**	1.066
**γ / °**	108.854(3)	**Final R indexes [I >=2σ (I)]**	R_1 = 0.0521, wR_2 = 0.1186

Volume / Å^3	471.17(7)	**Final R indexes (all data)**	R_1 = 0.0620, wR_2 = 0.1231
**Z**	1	**Largest diff. peak /hole**	0.29/-0.25
**ρ_{calc} g/cm^3**	1.320	**Flack parameter**	0.5(7)

Table S5. Hydrogen bonds for **G1** Form B.

D	H	A	d(D-H)/Å	d(H-A)/Å	d(D-A)/Å	D-H-A/°
N1	H1	O3[1]	0.81(5)	2.09(5)	2.845(5)	156(4)
N3	H3	O4[2]	0.93(5)	2.02(5)	2.899(4)	156(4)
N4	H4	O4[2]	0.97(7)	2.04(7)	2.947(4)	154(6)

[1]1+X,+Y,+Z; [2]-1+X,+Y,+Z

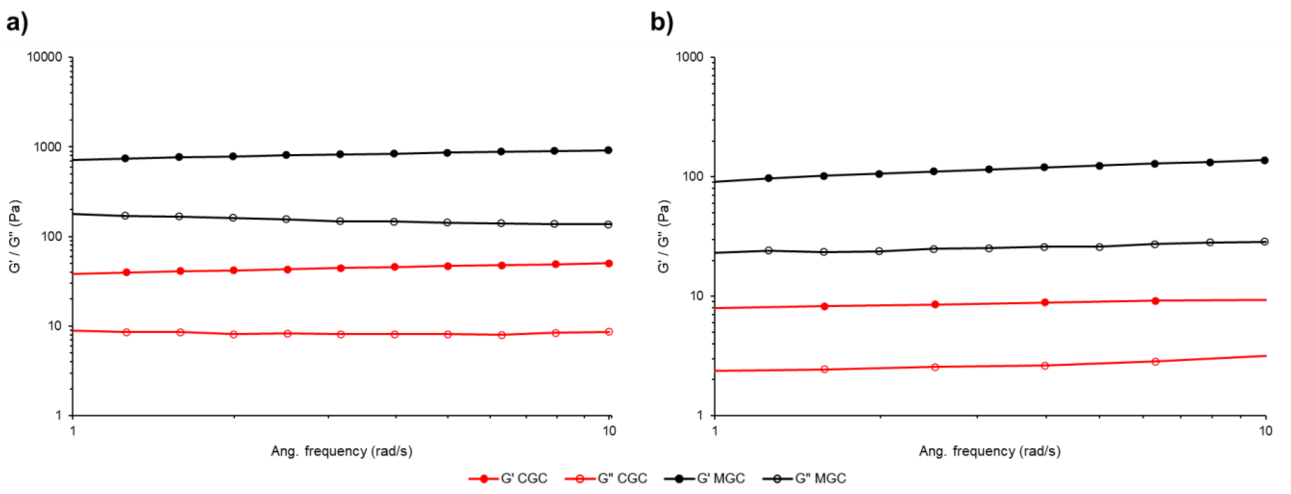

Figure S1. Frequency sweep rheology for a) **G1** and b) **G2** gels of cyclopentanone at both CGC and MGC, measured at a constant oscillatory stress of 0.5 Pa. Gels are characterized by a viscosity (slope of G' or G") that is invariant with angular frequency while at a constant oscillatory stress within the linear viscoelastic region (LVR).

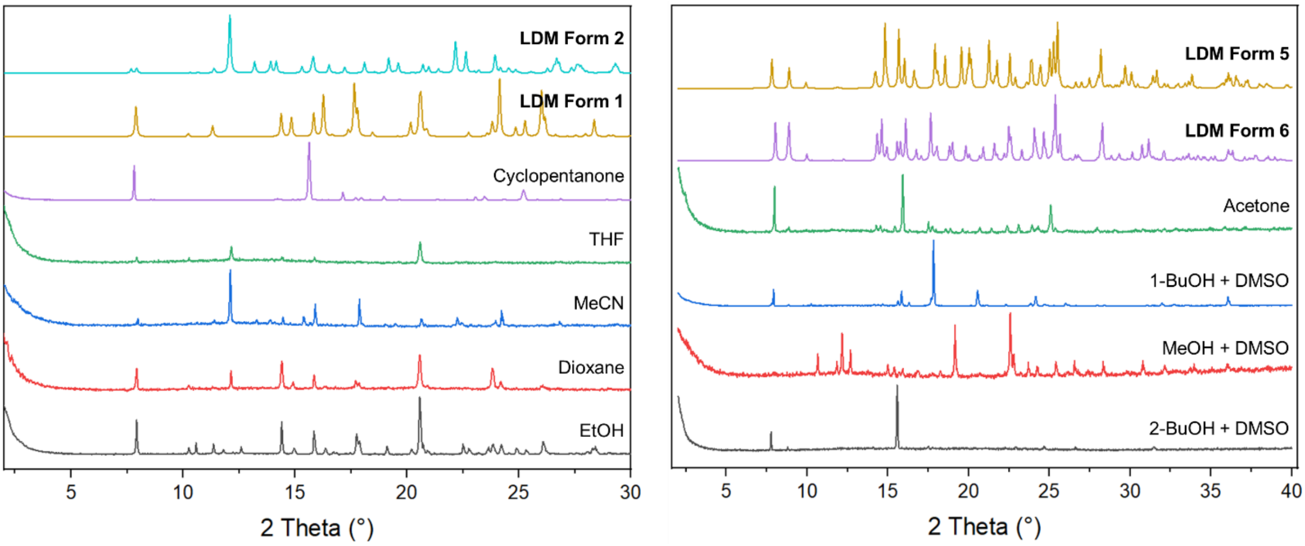

Figure S2. XRPD diffractograms for LDM recrystallisation experiments in solvents that can be gelled by gelator **G1** and/or **G2**. Powder patterns of known LDM polymorphs and solvates were simulated from SC-XRD data and shown in bold. This data was used in combination with single-crystal XRD to determine the polymorphic outcome from crystallisation in the absence of gelators **G1** and **G2**. The results are listed in Table 2.

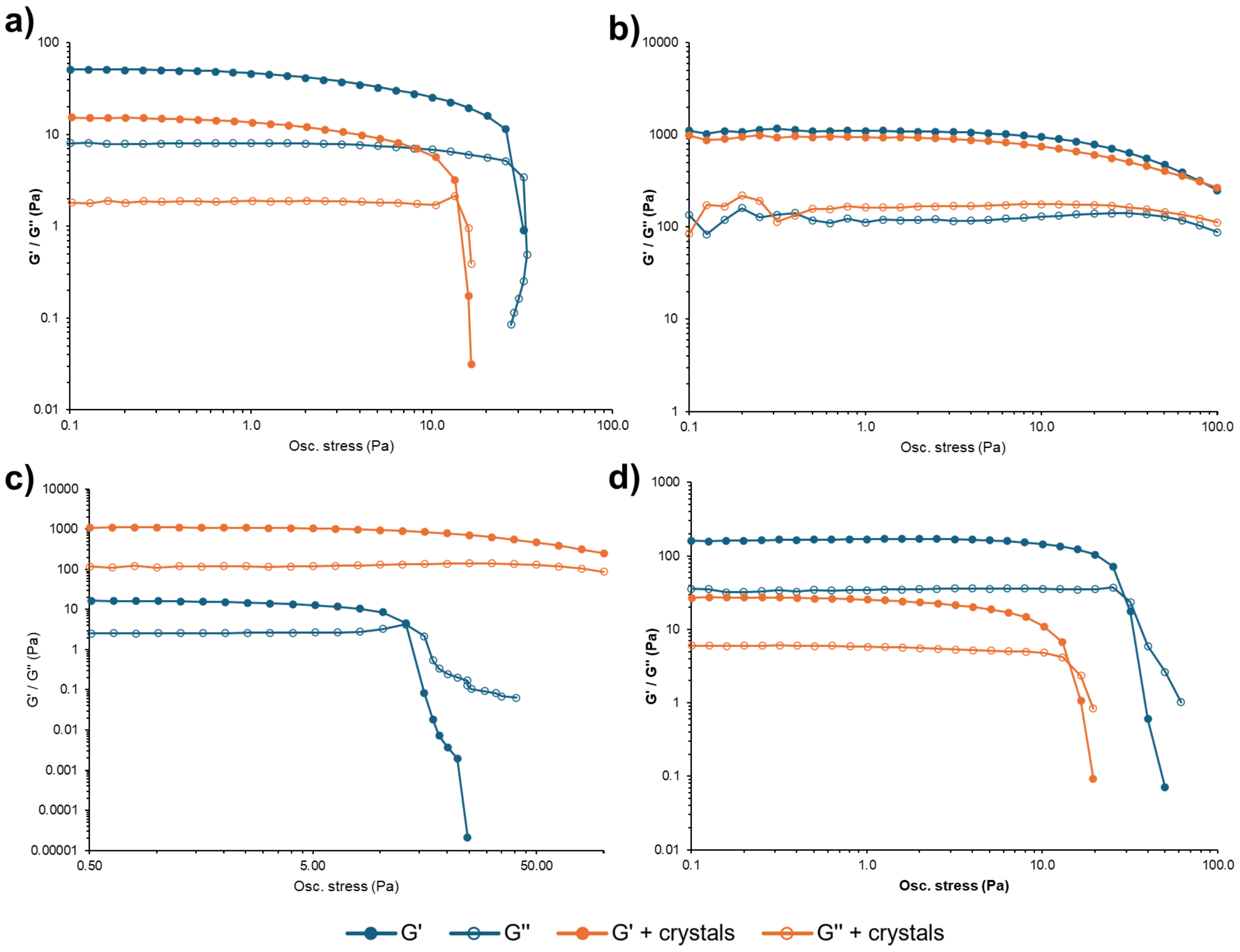

Figure S3. Oscillatory stress sweeps for cyclopentanone gels prepared with a) **G1** at CGC, b) **G1** at MGC, c) **G2** at CGC and d) **G2** at MGC, with and without the presence of LDM crystals. The presence of LDM crystals appears to weaken the gels produced with **G1** at CGC and **G2** at MGC, with a reduction in storage and loss moduli as well as a reduction in yield stress. By contrast, the gel produced with **G2** at CGC became stronger with a greater yield stress when LDM crystals were present. The **G1** gel at MGC appeared relatively unaffected by the presence of LDM crystals.

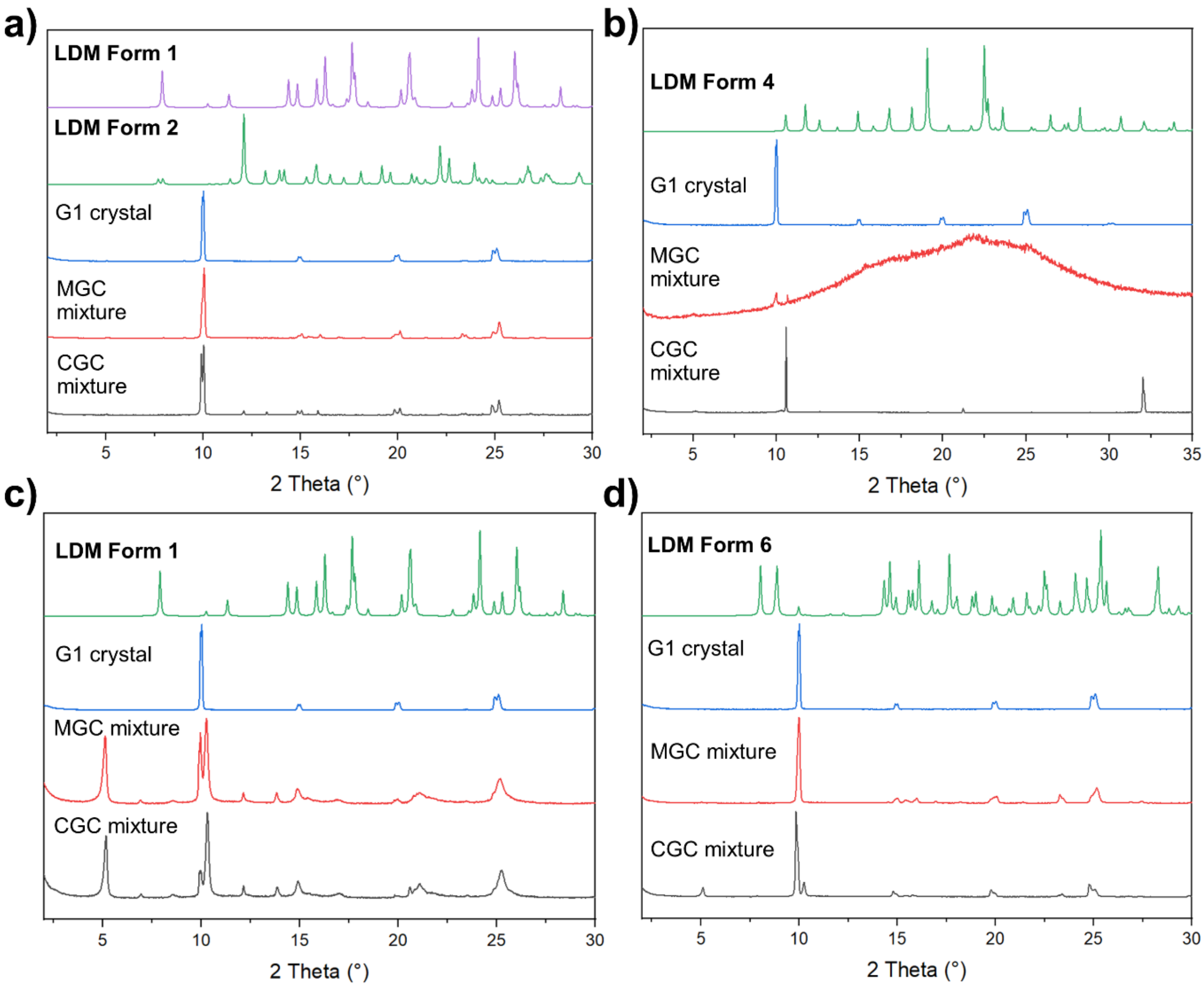

Figure S4. XRPD patterns of gel-crystal mixtures analysed from LDM recrystallisation experiments in **G1** gels of a) acetonitrile, b) nitrobenzene, c) tetrahydrofuran and d) acetone. Experiments at both CGC and MGC are compared. This data was used in combination with single-crystal XRD to determine the polymorphic outcome from crystallisation within **G1** gels. The powder pattern for LDM polymorphs and solvates were simulated from the SC-XRD data and shown in bold.

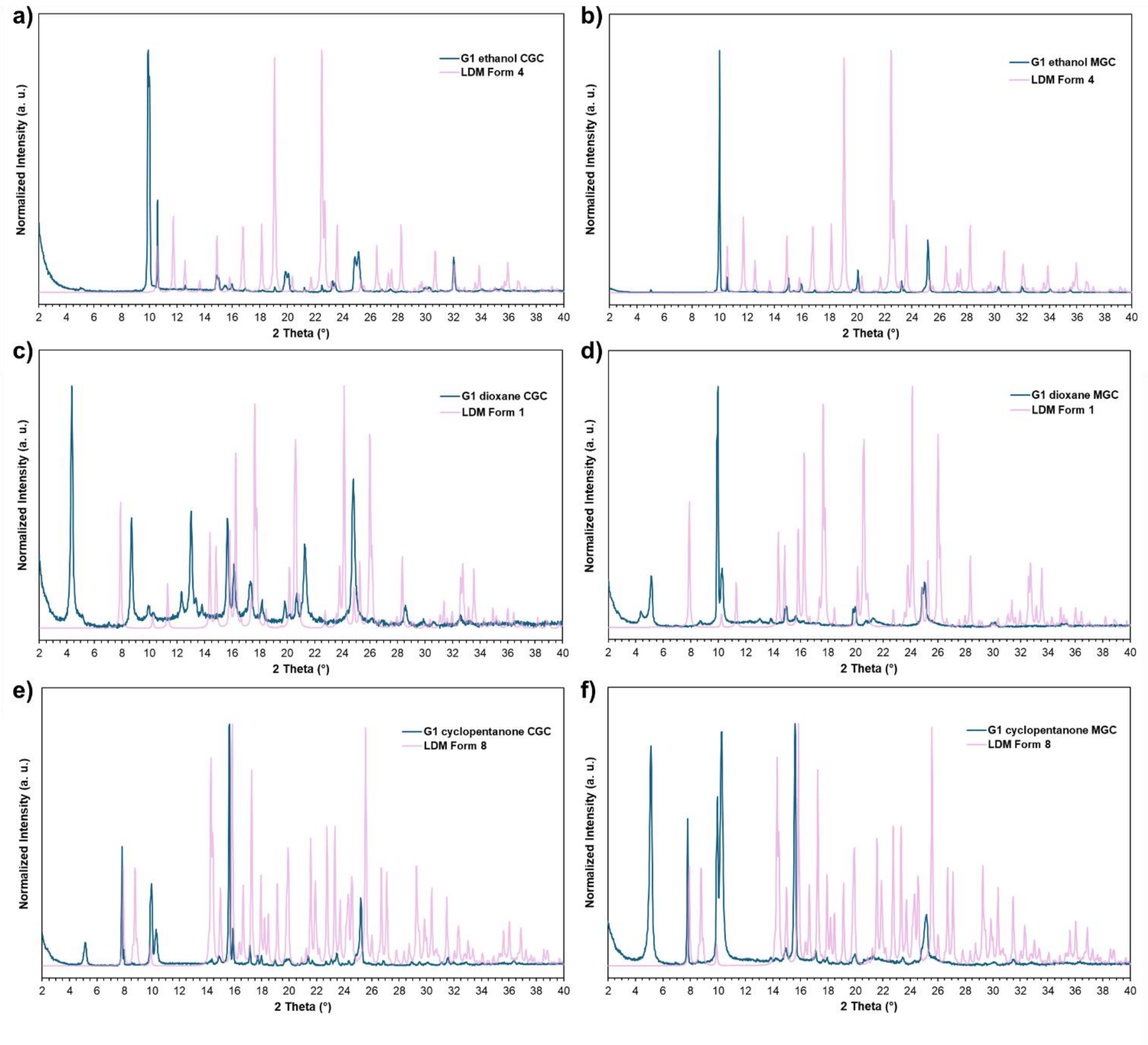

Figure S5. Overlays comparing experimental XRPD patterns (blue) of gel-crystal mixtures from LDM recrystallization experiments in **G1** gels of a-b) ethanol, c-d) dioxane and e-f) cyclopentanone with the patterns simulated from SC-XRD data (pink) of the LDM solid form identified in each recrystallization experiment via unit cell determination. Both experiments at CGC and MGC are shown for each solvent. Peaks from the simulated patterns may be missing in the experimental patterns due to preferred orientation effects, or because the LDM crystal peaks are obscured or drowned out by the **G1** xerogel peaks.

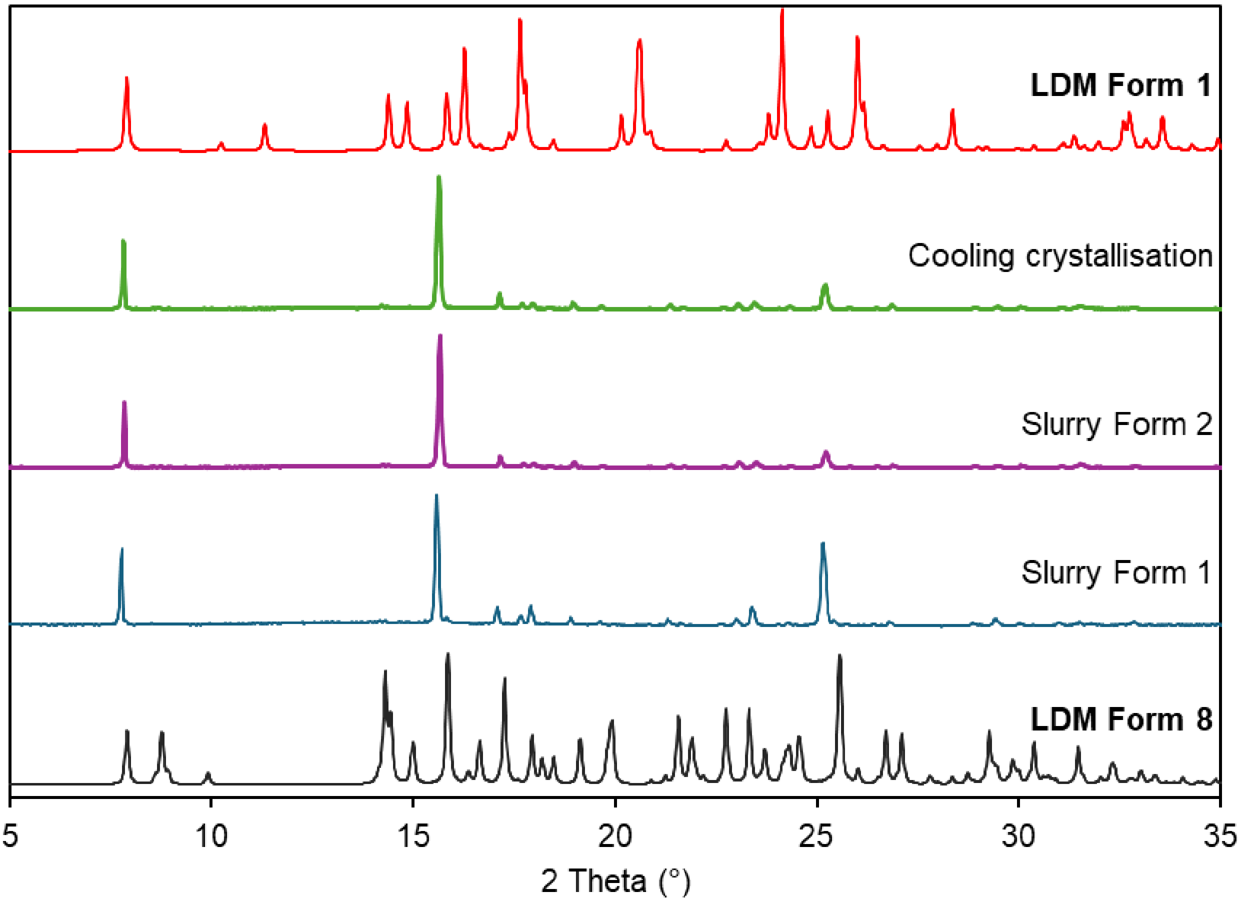

Figure S6. XPRD patterns for cooling and slurry crystallization experiments of LDM in cyclopentanone compared to the powder patterns of LDM Forms 1 and 8 simulated from SC-XRD data. Slurry experiments were conducted for 2 weeks starting from LDM Forms 1 and 2. Unit cell determination by SC-XRD confirmed that all three samples contained LDM Form 1.

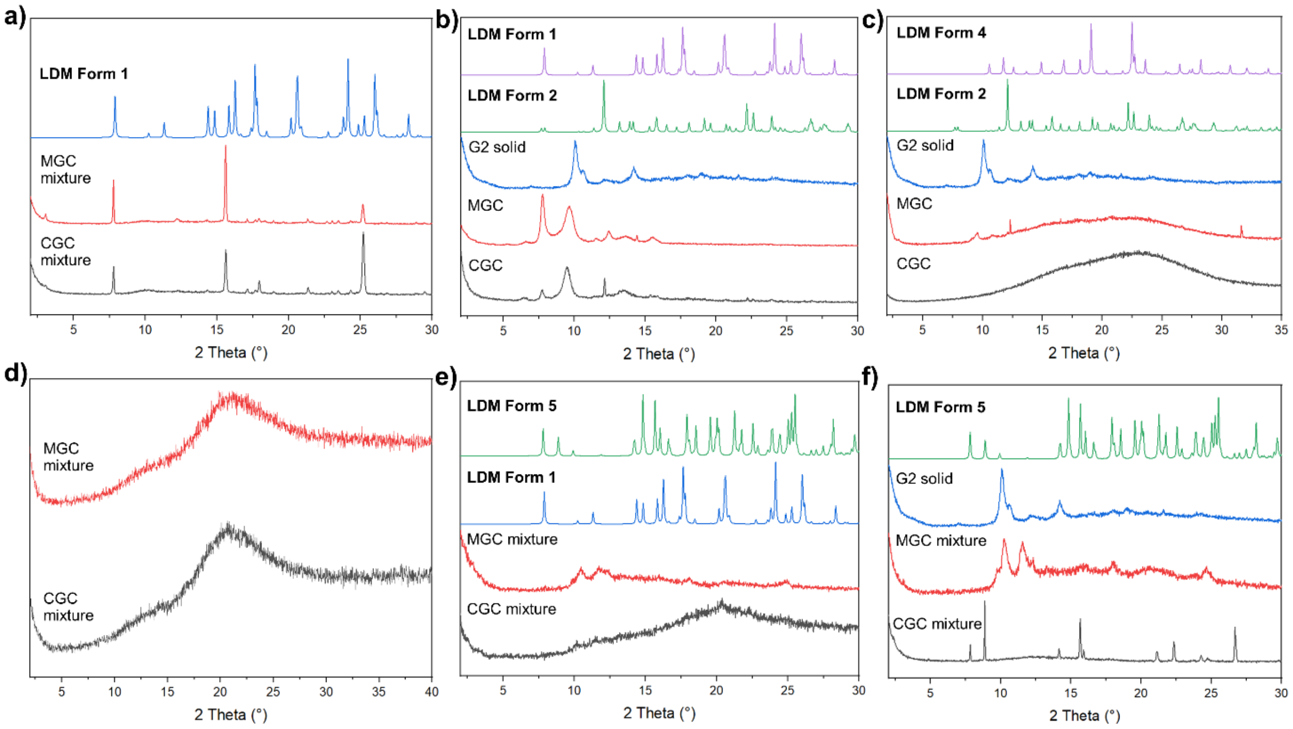

Figure S7. XRPD patterns of gel-crystal mixtures analysed from LDM recrystallisation experiments in **G2** gels of a) cyclopentanone, b) dioxane, c) nitrobenzene, d) methanol + DMSO, e) 1-butanol + DMSO and f) 2-butanol + DMSO. Experiments at both CGC and MGC are compared. This data was used in combination with single-crystal XRD to determine the polymorphic outcome from crystallisation within **G2** gels. The powder pattern for LDM polymorphs and solvates were simulated from the SC-XRD data and shown in bold.

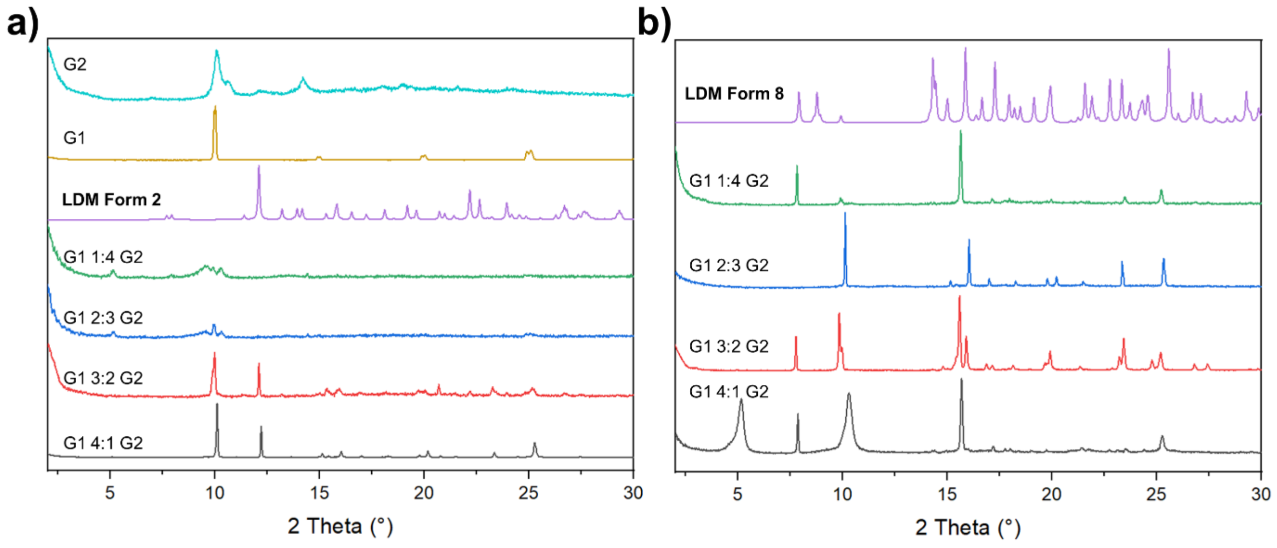

Figure S8. XRPD patterns of gel-crystal mixtures analysed from LDM recrystallisation experiments in gel blends of **G1** and **G2** at varying molar ratios in a) dioxane and b) cyclopentanone.

Table S6. Polymorphic outcome of LDM from recrystallisation experiments in gel blends of **G1** and **G2** at varying molar ratios in dioxane and cyclopentanone, determined by a combination of XRPD and SC-XRD.

**Dioxane**		**Cyclopentanone**	
Molar ratio G1:G2	LDM polymorphic outcome	Molar ratio G1:G2	LDM polymorphic outcome
1:4	Poor crystals	1:4	Form 8
2:3	Poor crystals	2:3	Form 8
3:2	Form 2	3:2	Form 8
4:1	Form 2	4:1	Form 8

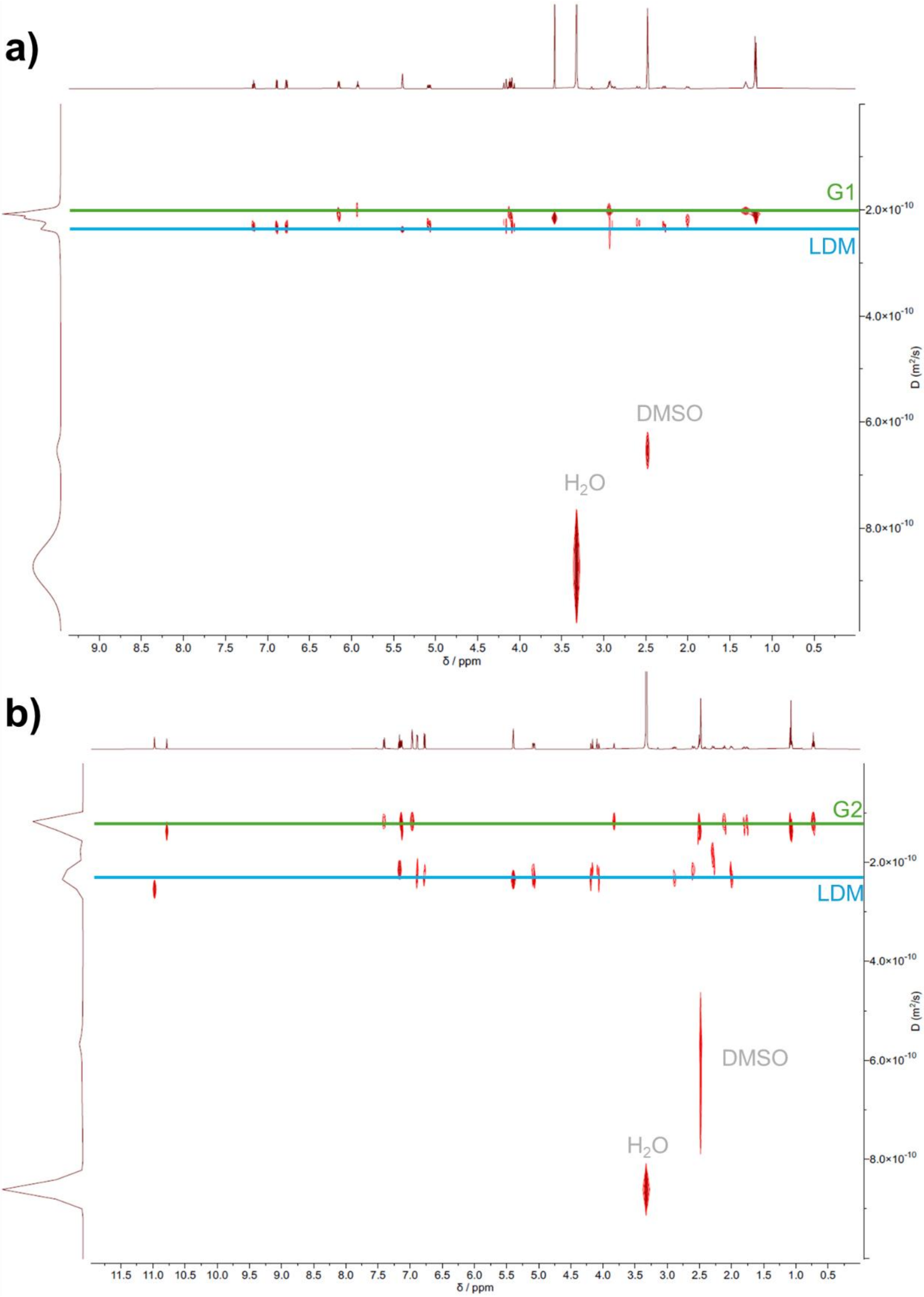

Figure S9. DOSY NMR spectra for LDM and a) **G1** or b) **G2** at a 1:1 molar ratio in DMSO-d_6, in which both drug and gelator components are fully soluble. In both cases, the drug and

gelator signals have different diffusion constants, indicating that they are not strongly interacting in solution in DMSO.

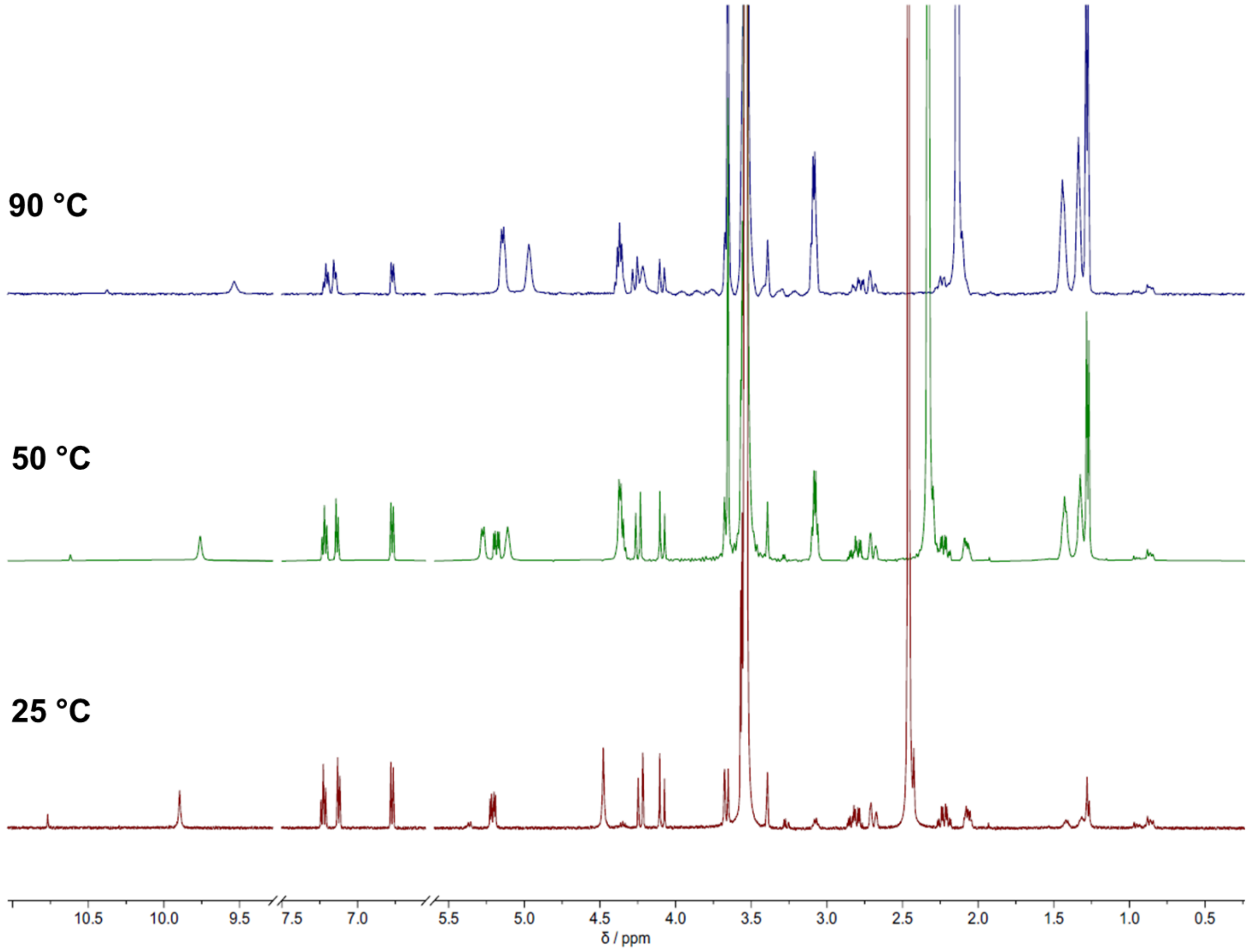

Figure S10. The ^{1}H NMR spectrum of a **G1** gel of dioxane-d8 at 1.5 % w/v containing 0.9 % w/v of LDM at 25 °C, 50 °C and 90 °C. As the gelatinous sample is warmed up and dissolves, the signals corresponding to **G1** increase in intensity while the LDM signals do not change in integral relative to the residual DMSO-d_5 solvent peak, indicating that no significant quantity of LDM molecules are incorporated into the **G1** gel fibres.
